# Transcriptome-Guided Functional Analyses Reveal Novel Biological Properties and Regulatory Hierarchy of Human Embryonic Stem Cell-Derived Ventricular Cardiomyocytes Crucial for Maturation

**DOI:** 10.1371/journal.pone.0077784

**Published:** 2013-10-21

**Authors:** Ellen Poon, Bin Yan, Shaohong Zhang, Stephanie Rushing, Wendy Keung, Lihuan Ren, Deborah K. Lieu, Lin Geng, Chi-Wing Kong, Jiaxian Wang, Hau San Wong, Kenneth R. Boheler, Ronald A. Li

**Affiliations:** 1 Stem Cell & Regenerative Medicine Consortium, LKS Faculty of Medicine, University of Hong Kong, Hong Kong, China; 2 Department of Physiology, LKS Faculty of Medicine, University of Hong Kong, China; 3 Department of Biology, Hong Kong Baptist University, Hong Kong, Hong Kong, China; 4 Department of Computer Science, City University of Hong Kong, Hong Kong, China; 5 Department of Computer Science, Guangzhou University, Guangzhou, China; 6 Center of Cardiovascular Research, Mount Sinai School of Medicine, New York, New York, United States of America; 7 Department of Internal Medicine, Division of Cardiovascular Medicine, University of California Davis, Davis, California, United States of America; 8 Division of Cardiology, Johns Hopkins University, Baltimore, Maryland, United States of America; Queen's University Belfast, United Kingdom

## Abstract

Human (h) embryonic stem cells (ESC) represent an unlimited source of cardiomyocytes (CMs); however, these differentiated cells are immature. Thus far, gene profiling studies have been performed with non-purified or non-chamber specific CMs. Here we took a combinatorial approach of using systems biology to guide functional discoveries of novel biological properties of purified hESC-derived ventricular (**V**) CMs. We profiled the transcriptomes of hESCs, hESC-, fetal (hF) and adult (hA) **V**CMs, and showed that hESC-**V**CMs displayed a unique transcriptomic signature. Not only did a detailed comparison between hESC-**V**CMs and hF-**V**CMs confirm known expression changes in metabolic and contractile genes, it further revealed novel differences in genes associated with reactive oxygen species (ROS) metabolism, migration and cell cycle, as well as potassium and calcium ion transport. Following these guides, we functionally confirmed that hESC-**V**CMs expressed I_KATP_ with immature properties, and were accordingly vulnerable to hypoxia/reoxygenation-induced apoptosis. For mechanistic insights, our coexpression and promoter analyses uncovered a novel transcriptional hierarchy involving select transcription factors (GATA4, HAND1, NKX2.5, PPARGC1A and TCF8), and genes involved in contraction, calcium homeostasis and metabolism. These data highlight novel expression and functional differences between hESC-**V**CMs and their fetal counterparts, and offer insights into the underlying cell developmental state. These findings may lead to mechanism-based methods for in vitro driven maturation.

## Introduction

The innate regenerative capacity of the adult mammalian heart is insufficient to restore function damaged by myocardial injury or heart failure. The identification of stem or progenitor cells that produce cardiomyocytes (CMs) has raised the intriguing possibility of cell-based cardiac regenerative therapies. Given the self-renewing capacity of pluripotent stem cells and their ability to differentiate into the cardiac lineage [1-6], human (h) embryonic stem cells (ESC) and induced pluripotent stem cell (iPSC) represent a potentially unlimited source of renewable CMs. However, hESC/iPSC-derived CMs are most similar to those of fetal tissues, functionally and structurally, and do not fully recapitulate post-natal or adult phenotypes [5,7-10]. For instance, hESC/iPSC-CMs have spontaneous contractile activities, express low levels of *I*_K1_, and relatively high currents from *I*_NCX_ and *I*_*f*_ [11]. Sarco/endoplasmic reticulum (SR) function remains rudimentary, as the cells exhibit a modest Ca^2+^ transient with slow kinetics, moderate SR Ca^2+^ATPase (SERCA) levels, low and disorganized ryanodine receptors and delayed phospholamban (PLN) expression [8,12] without the T-tubule system [9]. To overcome these limitations, an understanding of the molecular and cellular processes responsible for the development of a more physiological adult-like phenotype is required.

Microarray experiments have been performed to characterize the transcriptome of hESC-CMs and to identify signaling pathways implicated in their differentiation [13-17]. However, most of these studies were complicated by the presence of non-CMs in the cardiac biopsies and the use of un-staged and pooled fetal or adult heart samples [17,18] and non-purified or only partially purified ESC-CMs [13,14] without distinguishing the chamber-specific types. The expression data generally indicate that hESC-CMs express cardiac-specific genes, have lower levels of contractile and metabolic genes, and show differential expression of specific potassium/calcium ion channels, consistent with and complementary to known functional data. However, further functional analysis was often not performed and mechanistic insights into the causes of the immature state were lacking. 

By studying a purified population of hESC-derived ventricular (**V**) CMs (hESC-**V**CMs), as identified by the expression of a reporter under the transcriptional expression of the MLC2v promoter [19] and functionally confirmed by electrophysiological assays, here we took a combinatorial approach of using systems biology to guide functional discoveries of novel biological properties of hESC-**V**CMs. We performed microarray and bioinformatics analyses of staged human (h) fetal (F, 18-20 weeks), adult (A) and hESC-derived **V**CMs. As anticipated, our results show that hESC-**V**CMs have a unique transcriptomic signature, contractility and metabolic parameters that are most analogous to fetal cells; but, we also discovered a range of novel changes in cell cycle, reactive oxygen species (ROS) metabolism and migration that have not been previously reported. Focused analysis on genes involved in potassium and calcium ion transport further revealed novel functional immaturity. These results are discussed in relation to regulatory mechanisms.

## Materials and Methods

### Culture and isolation of undifferentiated hESCs and hESC-VCMs

Undifferentiated HES2 (ES02, ESI International, Singapore) were maintained at 37°C and 5% CO_2_ on irradiated mouse embryonic fibroblasts. Cardiac differentiation was performed using established protocols [20] (Methods S1). 20-30 days after differentiation, cells were transduced with recombinant LV-MLC2v-mCherry particles. Fluorescing mCherry-positive cells were isolated 72 hours post-transduction by flow-activated cell sorting (BD FACSAriaTM II). Resultant cells were >95% pure and displayed ventricular action potentials.

No ethical approval was required for preparing the mouse embryonic fibroblasts because the animals were sacrificed without any prior manipulation. Mice were sacrificed by pentabarbital.

### Isolation of hF- and hA-VCMs

HF-**V**CMs and hA-**V**CMs were isolated and experimented according to protocols approved by the UC Davis IUPAC and IRB (Protocol 200614787-1 and # 200614594-1) (Table S1, Methods S1).

### Transcriptomic Profiling

Cell samples were lysed in Trizol (Invitrogen). After adding 1:4 volume chloroform, aqueous and organic phases were separated. RNAs were extracted from the aqueous phase using the miRNeasy kit (Qiagen, Valencia, CA). Sentrix 48kb WG-6 beadchips (Illumina, San Diego, CA) were used to profile mRNA expression. Microarray data were analyzed using the BeadStudio transcriptomic (Illumina) software packages. (Please see methods S1 for details) 

### Co-expression and promoter analyses

Pearson correlation coefficient (PCC) was used to compare expression pattern across four groups of samples, hESCs, hESC-**V**CMs, hF-**V**CMs and hA-**V**CMs (PCC>0.95). We focused on 17 TFs including *FOXP1, GATA4, GATA6, HAND1, HAND2, IRX4, IRX5, ISL1, MEF2C, MESP1, NKX2-5, PPARA, PPARGC1A, SRF, TBX20, TBX5*, which have all been shown to play important roles in the heart, as well as *TCF8*. Binding sites of TFs GATA4, HAND1, NKX2-5 and TCF8 were predicted using Genomatix MatInspector. The promoter region between 1000 bp upstream and 300 bp downstream of 27 genes in the co-expression network was analyzed.

### Hypoxia treatment and TUNEL assay

HESC-**V**CMs of 20-30 days post-differentiation were subjected to hypoxia (1% O_2_)/reoxygenation for different durations as stated. For hypoxia, hESC-**V**CMs were transferred to a CO_2_ incubator with 95% N_2_/5% CO_2_ at 37°C. After hypoxia, cells were transferred back to 95% air/5% CO_2_ for reoxygenation. TUNEL assay was performed as per manufacturer’s instructions (Roche Diagnostics).

### Electrophysiology

Electrical recordings were performed using the whole-cell patch-clamp technique as previously described [21,22] at 37°C. For *I*_KATP_ measurements, the internal pipette solution contained (mM) potassium glutamate 120, KCl 25, ATP (magnesium salt) 1, EGTA 10, MgCl_2_ 0.5, and HEPES 10 (pH 7.2). The external bath solution contained (mM) NaCl 140, KCl 5, MgCl_2_ 1, CaCl_2_ 1, and HEPES 10 (pH 7.4) [21]. To induce hypotonic stress, a modified Tyrode’s solution consisting of 64mM NaCl and 50mM mannitol was superfused with a flow speed of 2ml/min to replace the control solution consisting of 64mM NaCl and 150mM mannitol. 

### Optical Mapping

Transmembrane potential of hESC-VCM monolayers was optically mapped by using MiCam Ultima (Scimedia, USA) fluorescence non-contact imaging system with a 1cm^2^ field-of-view. Briefly, hESC-VCM preparations, which were cultured on gelatin-coated glass coverslips, were incubated with 4-8µM di-4-ANEPPS (Invitrogen, USA) for 20 min at room temperature in Tyrode’s solution, consisting of (mM) 140 NaCl, 5 KCl, 1 MgCl_2_, 1 CaCl_2_, 10 D-glucose and 10 HEPES at pH 7.4. Monolayers were rinsed twice with pre-warmed (37°C) Tyrode’s solution before imaging using a halogen light, which was filtered by a 515±35 nm band-pass excitation filter and a 590 nm high-pass emission filter. A co-axial point stimulation electrode was used to deliver a steady-state pacing at 1 Hz, 8V and 10ms pulse duration. Please see methods S1 for more details.

## Results

### Isolation and characterization of hESC-VCM

**Figure 1:**
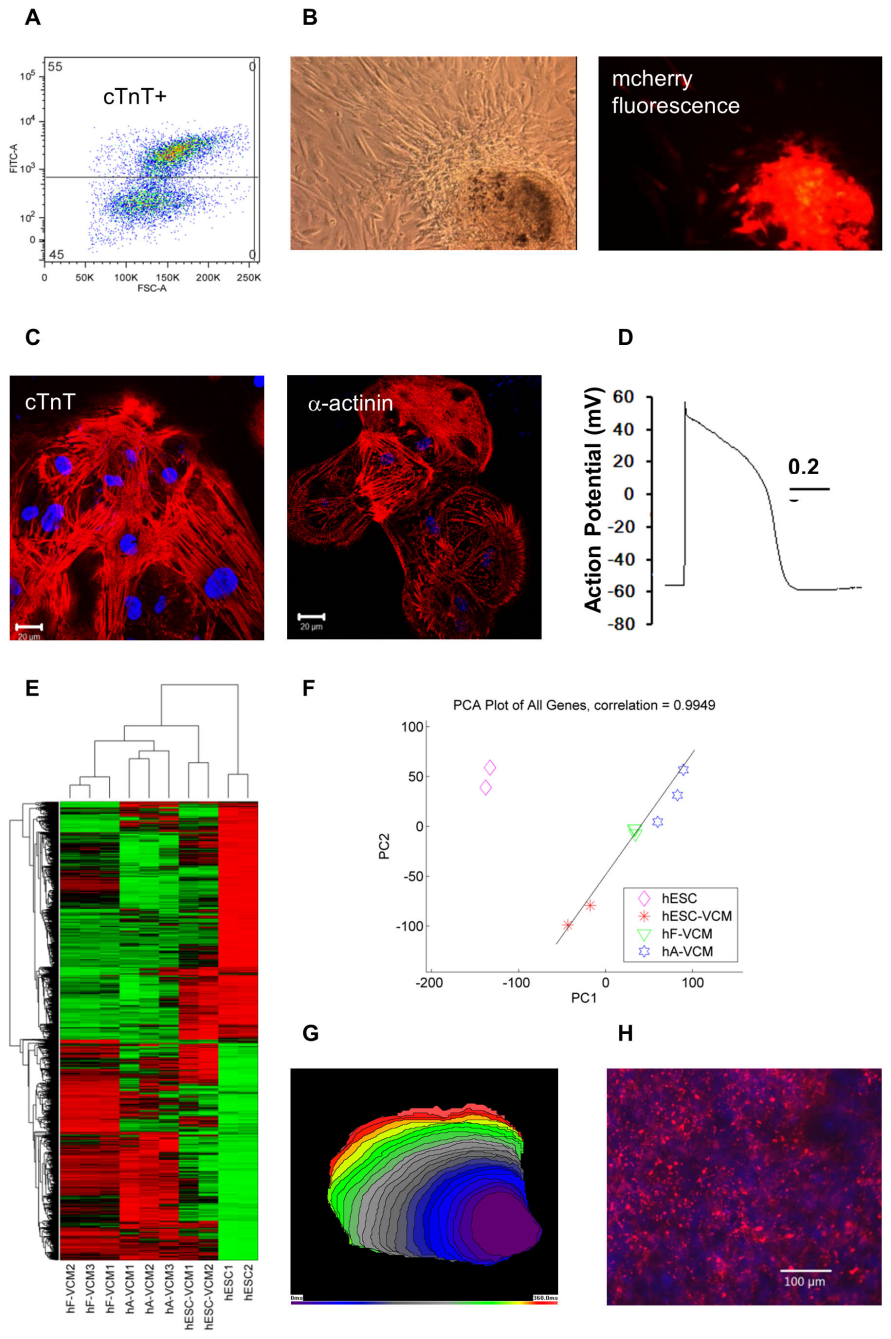
Developmental classification of hESC-VCMs. A) A representative FACS plot showing that approximately 55% of hESC-derivatives were positive for cardiac troponin-T (cTnT) prior to selection. B) Selected MLC2V-mCherry+ hESC-**V**CMs (~95% purified) under brightfield and fluorescence microscopy (100x). C) Purified HESC-**V**CMs were stained with anti-cTnT and anti-α-actinin antibodies. D) Representative action potential of hESC-**V**CMs. E) Hierarchical clustering showing that biological replicates cluster together. Red and green indicates up- and down-regulation respectively. F) Principal Component Analysis. HESC-**V**CMs, hF-**V**CMs and hA-**V**CMs lie along a linear developmental axis (dotted black line). Correlation coefficient is shown to indicate the degree of linearity. G) An activation isochronal map of trans-membrane potential with 15ms intervals. H) CX43 staining of hESC-**V**CMs. Red= CX43 staining, blue=DAPI.

We performed directed cardiac differentiation as described previously [20] with cardiac derivatives making up ~50% of the cell population (Figure 1A). HESC-**V**CMs were identified by the expression of a reporter under the transcriptional control of the MLC2v promoter [19] (Figure 1B) and were isolated by flow-activated cell sorting. Resultant cells were >95% pure (Figure 1C) and displayed ventricular action potentials (Figure 1D). 

### HESC-VCMs displayed a unique transcriptional signature and were less developmentally advanced than hF-VCMs

We performed transcriptomic analyses of microarrays containing 48,804 probes corresponding to 25440 genes. Pluripotency genes such as *POU5F1*(Oct4) and *NANOG* were highly expressed in hESCs but absent in all of hESC-, hF- and hA-**V**CM samples. Conversely, cardiac markers such as *MYL2*, *TNNT2* and *ACTN2* were specifically detected in the three **V**CM populations, confirming their cardiac identities. Hierarchical clustering and principal components analysis (PCA) established that overall gene expressions among biological replicates were very similar (Figure 1E and F). The transcriptomic expression of hESC-**V**CM samples grouped more similarly to hF-**V**CMs and hA-**V**CMs than to hESCs (Figure 1F). The most conspicuous trend in global gene expression was that genes of hESC-**V**CMs, hF-**V**CMs and hA-**V**CMs on the PCA plot followed a virtually linear relationship (Figure 1F) with hESC-**V**CM, hF-**V**CM, and hA-**V**CM group of genes situated at the bottom left, middle and top right, respectively, which is consistent with a progressive increase in cellular maturity. A comparison between hESC-**V**CMs and hF-**V**CMs showed that 1852 (7%) and 2195 (9%) genes were up-/down-regulated by more than 2-fold in hESC-**V**CMs relative to hF-**V**CMs respectively.

### HESC-VCMs expressed significant levels of chamber-specific genes similar to chamber myocardium, but with a slower conduction velocity than hF-VCMs

We further assessed the developmental status of hESC-**V**CMs by the use of molecular markers that are restricted to or upregulated in chamber myocardium relative to the primitive myocardium in human [23] and mice [24]. We detected significant gene expression from *NPPA* (atrial natriuretic factor)*, GJA1* (gap junction alpha-1/connexin 43)*, GJA5* (gap junction alpha-5/connexin 40)*, SMPX*(chisel) and *IRX5*(Iroquis-5) (p<0.05) in hESC-**V**CMs; however, the expression of *GJA5, SMPX*, and *IRX5* in hESC-**V**CMs was 4.7-, 3.2-, and 2.4- fold lower than those in hF-**V**CMs. The data showed that the hESC-**V**CMs displayed gene expression profiles typical of early chamber myocardium, suggesting that the cells were less developmentally advanced than the hF-**V**CMs analyzed in this study. The general immaturity of hESC-**V**CMs and more specifically the reduced expression of *GJA5*, is also typical of very immature chamber myocardium, which is known to have a very slow conduction velocity. Consistently, optical mapping of purified hESC-**V**CM in monolayer culture confirmed that the conduction velocity (5.0±1.4 cm/s) was much slower than that of the intact adult human heart (7-80 cm/s depending on fibre direction) [25] (Figure 1G) but is similar to that of non-purified hESC-CMs (5±6 cm/s) [11]. Furthermore, CX43-positive gap junctions are aligned at the intercalated disc of adult CMs [26] but such was not the case for hESC-**V**CMs, whose CX43-positive gap junctions were randomly distributed (Figure 1H). Evidently, conduction pattern was isotropic as opposed to anisotropic as observed in the native myocardium [27].

### Examination of the most abundant transcripts in CM samples identified novel markers of cardiac maturation

**Figure 2:**
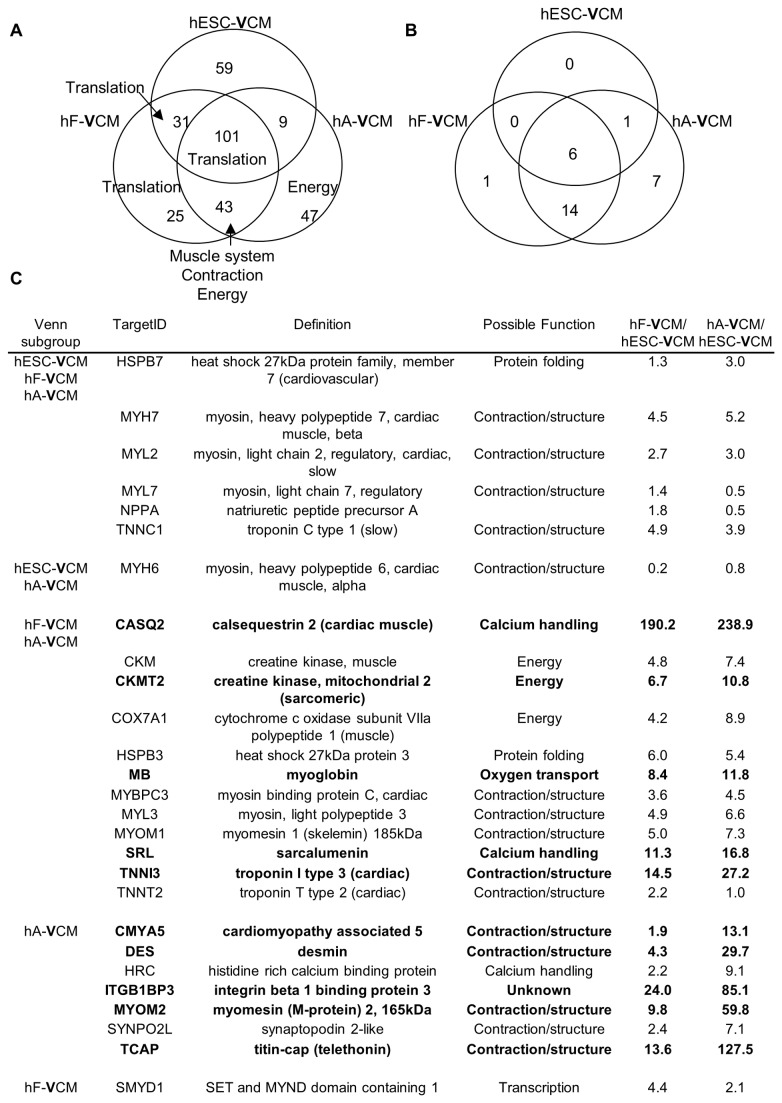
The most abundant transcripts in VCM populations. A) The Venn diagram shows the distribution of the top 200 most abundant genes in hESC-**V**CMs, hF-**V**CMs and hA-**V**CMs. The number of genes in common is indicated. The labels refer to biological processes that are enriched. B) The tissue-specific distribution of the top 200 genes was examined in public databases including Genenote and bioGPS. The ‘cardiac-specific’ genes among the top 200 most abundant genes are shown. C) 27 cardiac-specific genes within the top 200 most abundant genes in hESC-**V**CMs, hF-**V**CMs and/or hA-**V**CMs. Fold change relative to hESC-VCMs are indicated and fold change >10 is highlighted in bold. Possible function is based on gene ontology association.

The functions of the 200 most abundant transcripts in hESC-, hF- and hA-**V**CMs were examined by Gene Ontology analysis (Table S2). The three populations showed a high level of similarity, with 101 common transcripts involving mostly translation elongation (Figure 2A). HA- and hF-**V**CMs shared 43 transcripts enriched for muscle system, contraction and energy generation, while hESC- and hF-**V**CMs shared only 31 involved in translational elongation. Transcripts involved in energy generation were particularly abundant in hA-**V**CMs, consistent with their high metabolism. We focused on heart-specific genes to identify novel markers for CM identity and maturation. Twenty-seven gene products were more than 10-fold enriched in the heart and skeletal muscle, including known cardiac markers such as *MYL2, MYH7 and TNNC1* (Figure 2B and C). Six genes (*HSPB7, MYH7, MYL2, MYL7, NPPA* and *TNNC1*) were within the top 200 abundant genes in all three CM samples and may serve as markers of cardiac identity. Ten gene transcripts were more than 10-fold depleted in hESC-**V**CMs relative to hF-**V**CMs and/or hA-**V**CMs. Of these, the expression of *CKMT2, SRL, CMYA5, ITGB1BP3, MYOM2* and *TCAP* in hESC-**V**CMs have not been described previously. *CKMT2* is involved in energy generation. *CMYA5, MYOM2* and *TCAP* are structural genes while *SRL* encodes a Ca^2+^ binding protein. *ITGB1BP3* encodes an integrin beta 1 binding protein with unknown function in the heart.

### Gene Set Enrichment Analysis (GSEA) reveals novel expression differences in cell cycle, ROS metabolism and migration

GSEA was employed to identify specific expression differences between hF-**V**CMs and hESC-**V**CMs. We examined 1403 gene sets among 25440 genes and found that 119 (8%) and 572 (41%) gene sets were significantly decreased and increased in hESC-**V**CMs relative to hF-**V**CMs respectively. The 50 most differentially expressed gene sets are summarized in Table 1 and listed in Table S3A and S3B. QRT-PCR analysis was performed on selected genes to verify the microarray data (Figure S1).

**Table 1:** Summary of top 50 gene sets which showed differential expression between hESC-VCMs and hF-VCMs. Please refer to Table S3A and B for details.

hESC-**V**CM depleted	# gene sets	hESC-**V**CM enriched	# gene sets
Cell Cycle	20	Cytokine and defense	17
Metabolism	9	Stress	6
Contraction	6	Movement	11
ROS metabolism	2	Signaling	3
Heart development	4	Development	1
Telomere	2	Apoptosis	1
Others	7	Others	11

Consistent with previous reports, gene sets related to respiration, tricarboxylic acid (TCA) cycle, fatty acid oxidation and heart development were decreased in hESC-**V**CMs compared to hF-**V**CMs [17]. Of expression changes that have not been previously described, cell cycle-related gene sets showed the most significant decrease in expression in hESC-**V**CMs, with extremely low false discovery rate (FDR) scores of <0.0001. They were also the most abundant, totaling 20 out of 50 top most reduced gene sets. These gene sets encompassed various stages including G1-M phases and cytokinesis, suggestive of inhibition at multiple stages of the cell cycle. Cycling cells require mechanisms to preserve genomic integrity during DNA replication and we detected lower level of genes associated with telomere maintenance, consistent with the decreased level of cell cycle genes. Genes involved in ROS metabolism were down-regulated in hESC-**V**CMs. In particular, catalase (*CAT*) and glutathione peroxidase (*GPX3*) are important for ROS removal and both were found at low and significantly reduced levels (10- and 5-fold respectively) in hESC-**V**CMs compared to hF-**V**CMs. ROS plays an important role in the induction of apoptosis and expression of apoptotic genes were also enhanced in hESC-**V**CMs. 

There was an increased expression of genes associated with cell migration, in 11 out of the top 50 gene sets. Examples included pro-migratory proteases and receptors e.g., *THBS1, F2RL1*, and *SPHK1*, signaling molecules *TGFB2* and *BMP2*, and matrix metalloproteinase *MMP9*. Consistent with this migratory phenotype, we observed higher expression of genes important for epithelial-mesenchymal transformation. Increased expression of genes associated with extracellular matrix organization e.g., *COL2A1* was also noted although these latter changes were not within the top 50 differentially gene sets. Genes associated with cytokine stimulation and cellular defense were also up-regulated in hESC-**V**CMs.

### Novel expression changes in potassium and calcium transport genes

Even though the electrophysiological attributes of hESC-CMs are known to be immature [7-10,19], gene sets related to potassium and calcium ion transport were not differentially expressed between hESC-**V**CMs and hF-**V**CMs; however, specific genes did show significant differences. Among Ca^2+^ transport genes, *SLC8A1, PLN, RYR2, CACNB2, CAV3, CAMK2A* and *CACNA1C* levels were lower in hESC-**V**CMs, as previously reported [14,18]. We also observed novel changes in genes important for Ca^2+^ handling (Table 2). Sarcalumenin (*SRL*) is a SR protein thought to regulate SERCA stability and was reduced by 11.3-fold in hESC-**V**CMs. *CAMK2D* and *CAMK2B* encode Ca^2+^/Calmodulin kinases (CAMKII) and multiple isoforms of these genes were significantly reduced in hESC-VCMs. Phospholemman (*FXYD1*) modulates Na^+^/K^+^ ATPase, *I*_NCX_ and *I*_Ca_ and was also highly reduced. Other genes that were diminished included gap junction gene *GJA4* (4.7-fold), endothelin receptors, *EDNRA* (2.6-fold) and *EDNRB* (4.6-fold), and *CAV1* (2.8-fold) etc (Table 2). A comparison with hA-**V**CMs further showed that most of these genes (*CAMK2B, CAMK2D, SRL, FXYD1* and *ENDRB*) were expressed at increasing levels in hESC-, hF- and hA-**V**CMs, which supports their potential role in cardiac maturation. 

**Table 2:** Novel expression changes in genes involved in calcium ion transport and homeostasis, p<0.05.

Gene	hF-VCM/ hESC-VCM	Description	
SRL	11.3	Sarcalumenin
CAMK2B	8.1, 5.5, 4.5, 4.0*	calcium/calmodulin-dependent protein kinase II beta
FXYD1	7.6, 5.4, 2.6*	FXYD domain containing ion transport regulator 1 (phospholemman)
CACNB2	5.3	calcium channel, voltage-dependent, beta 2 subunit
GJA4	4.7	gap junction protein, alpha 4, 37kDa
EDNRB	4.6	endothelin receptor type B
CAMK2D	3.7, 2.5*	calcium/calmodulin-dependent protein kinase II delta
JAK2	3.3	Janus kinase 2 (a protein tyrosine kinase)
EPHX2	2.8	epoxide hydrolase 2, cytoplasmic
CAV1	2.8	caveolin 1, caveolae protein, 22kDa
KDR	2.7	kinase insert domain receptor
CSRP3	2.6	cysteine and glycine-rich protein 3 (cardiac LIM protein)
PLCG2	2.6	phospholipase C, gamma 2 (phosphatidylinositol-specific)
EDNRA	2.6	endothelin receptor type A

* Fold changes for multiple transcript variants of the same gene.

We also detected novel expression changes in genes encoding potassium channels, and consistent with previous results, significantly reduced levels of *KCNQ1, KCNE1, KCNAB1, KCNJ2* and *KCNJ8* were observed [14,18,28]. The novel changes in potassium channels reported here are implicated in cardioprotection and include genes encoding *I*_KATP_, *I*_KCa_ and *I*_KNa_ (Table 3). Transcript variants for the regulatory unit of *I*_KATP_, *ABCC9*, were either absent or reduced in hESC-**V**CMs compared to hF-**V**CMs. Isoform-specific expression of *I*_KCa_ (*KCNMB1* and *KCNMB3*) and *I*_KNa_ (*KCNT1* and *KCNT2*) subunits were also observed. *AQP1* (aquaporin-1) was decreased in hESC-**V**CMs. *KCNG1, KCTD8, FXYD2* and *KCNIP4* were uniquely expressed in hESC-**V**CMs.

**Table 3:** Novel expression changes in genes involved in potassium ion transport and homeostasis, p<0.05.

Gene	hF-**V**CM/ hESC-**V**CM	Description
KCNT1	hF-**V**CM only	potassium channel, subfamily T, member 1
ABCC9	hF-**V**CM only, 6.7, 6.6*	ATP-binding cassette, sub-family C (CFTR/MRP), member 9
KCNMB1	6.3	potassium large conductance calcium-activated channel, subfamily M, beta member 1
AQP1	3.0	aquaporin 1
CHP	2.1	calcium binding protein P22
NSF	0.5	N-ethylmaleimide-sensitive factor
KCNMB3	0.3	potassium large conductance calcium-activated channel, subfamily M beta member 3
NSF	0.2	N-ethylmaleimide-sensitive factor
KCTD8	hESC-**V**CM only	potassium channel tetramerisation domain containing 8
KCNT2	hESC-**V**CM only	potassium channel, subfamily T, member 2
KCNG1	hESC-**V**CM only	potassium voltage-gated channel, subfamily G, member 1
FXYD2	hESC-**V**CM only	FXYD domain containing ion transport regulator 2
KCNIP4	hESC-**V**CM only	Kv channel interacting protein 4

* Fold changes for multiple transcript variants of the same gene.

### Cardioprotective mechanisms in hESC-VCMs

**Figure 3:**
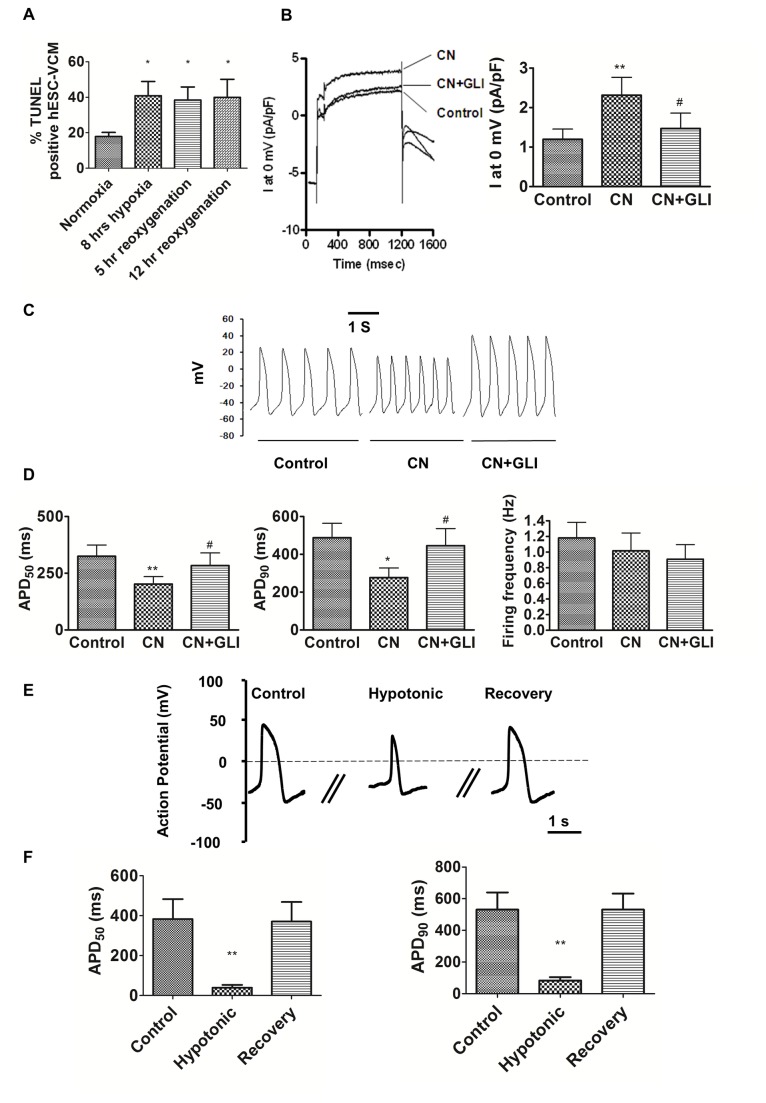
Functional analyses of hESC-VCMs. A) Apoptotic response after hypoxia/reoxygenation was assessed using TUNEL assay. HESC-**V**CMs were subjected to hypoxic treatment, followed by reoxygenation of different duration as indicated. *P<0.05 relative to normoxic conditions. B) *I*_KATP_ channel current and its effect on electrophysiological properties of hESC-**V**CMs. Representative current and summary of current densities of whole-cell patch-clamp recording of hESC-**V**CMs. Extracellular application of *I*_KATP_ channel openers, 2 mmol/L sodium cyanide (CN), at 2 min significantly increased current densities compared to controls, which was inhibited by *I*_KATP_ channel blocker 10 uM glibenclamide (GLI). Cells were stimulated to 0 mV for 1000 ms from a holding potential of -80 mV preceded by a 100-ms prepulse to -10 mV. C) Representative tracings of action potentials of hESC-**V**CMs before and after treatment of 2 mM CN and then 10 uM GLI. D) The effect of CN and GLI on APD50, APD90 and firing frequency. N=6; *P<0.05, **P<0.01 compared to control group; #P<0.05, compared to CN group. Effect of hypotonic insult on hESC-**V**CMs. E) Representative tracings of action potentials of hESC-**V**CMs before and after hypotonic insult, and upon recovery. F) The effect of hypotonic insult on APD50 and APD90. N=6; **P<0.01 compared to control group.

*I*_KATP_, *I*_KCa_, *I*_KNa_, ROS metabolic enzymes and AQP1 are important for cardioprotection by maintaining cellular homeostasis. We postulated that reduced/perturbed expression would result in increased susceptibility to injury. To test this, we subjected hESC-**V**CMs to hypoxia and hypoxia/reoxygenation, which is a model for ischemia/reperfusion injury. Consistent with our postulate, we observed a 2-fold increase in TUNEL positive apoptotic cells after hypoxia and hypoxia/reoxygenation compared with normoxic conditions (Figure 3A). By contrast, hF-**V**CMs showed almost no increase in cell death after a similar protocol [29]. 

To further explore the mechanisms underlying this increased cell vulnerability, we focused on the functionality of *I*_KATP_, which regulates ion homeostasis in response to ATP depletion during ischemic insult, and showed that *I*_KATP_ was reduced in hESC-**V**CMs. Sodium cyanide (CN) can induce ATP depletion via uncoupling of oxidative phosphorylation. Application of CN induced an outward current and significantly increased current densities from 1.2+/-0.3 to 2.3+/-0.5 pA/pF, which was inhibited by *I*_KATP_ blocker glibenclamide (GLI) (Figure 3B). Of note, *I*_KATP_ in hESC-**V**CMs was smaller than that reported for hA-**A**CMs (7.3 ± 2 pA/pF) [30] and mouse adult CMs (approx. 23 pA/pF) [21]. Figure 3C further shows the role of *I*_KATP_ in the AP waveform. CN significantly reduced APD50 and APD90 to 61.0 and 56.7% of control, thereby hastening the spontaneous firing (Figure 3D). CN had no significant effect on AP amplitude or frequency (Figure 3D). This CN-mediated AP shortening could be abolished by blockade of *I*_KATP_ by GLI. In addition to ATP depletion, ischemic insult is also accompanied by a cytosolic buildup of metabolites, increased intracellular osmolarity, followed by cell swelling. Given that *I*_KATP_ and aquaporin-1 are both involved in osmotic regulation, and their reduced expression in hESC-**V**CMs may result in perturbed responses to hypertonic stress (which simulates ischemic injury), we tested the effect of hypotonic treatment on hESC-**V**CMs [22]. We found that hypotonic stress indeed resulted in the loss of spontaneous AP in hESC-**V**CMs. 1000pA-5ms stimulation produced an AP with significantly reduced APD50 and APD90 10.1% and 15.6% of control (Figure 3E and F). Upon recovery, AP returned to pre-treatment conditions. Thus hESC-**V**CMs were able to undergo AP shortening upon hypotonic treatment, as was reported for adult guinea pig CMs [22]. 

### Cardiac transcription factors: expression, co-expression and promoter analyses

To unravel possible regulatory mechanisms for the developmental immaturity of hESC-**V**CMs, we analyzed the expression of transcription factors (TFs) critical for heart development. We found that transcripts from *TBX5* (12.9 fold), *TBX20* (6.9 fold), *GATA4* (1.3 fold) and *IRX5* (2.4 fold), which are implicated in chamber formation [31-33], were reduced in hESC-**V**CMs. Similarly, transcripts encoding critical cardiac developmental TF genes, including *HAND2* (3.0 fold), *GATA6* (3.0 fold), *IRX4* (2.6 fold), *SRF* (2.5 fold), were significantly decreased. Conversely, *TBX2* [34] and *TBX3* [35] locally repress chamber differentiation and *TBX3* was 3-fold enriched in hESC-**V**CMs. The expression of transcription factors *ISL1*, *MEF2C, MESP1, FOXP1, HAND1, NKX2.5* and *PPARGC1A* were not significantly different between hESC-**V**CMs and hF-**V**CMs. 

**Figure 4:**
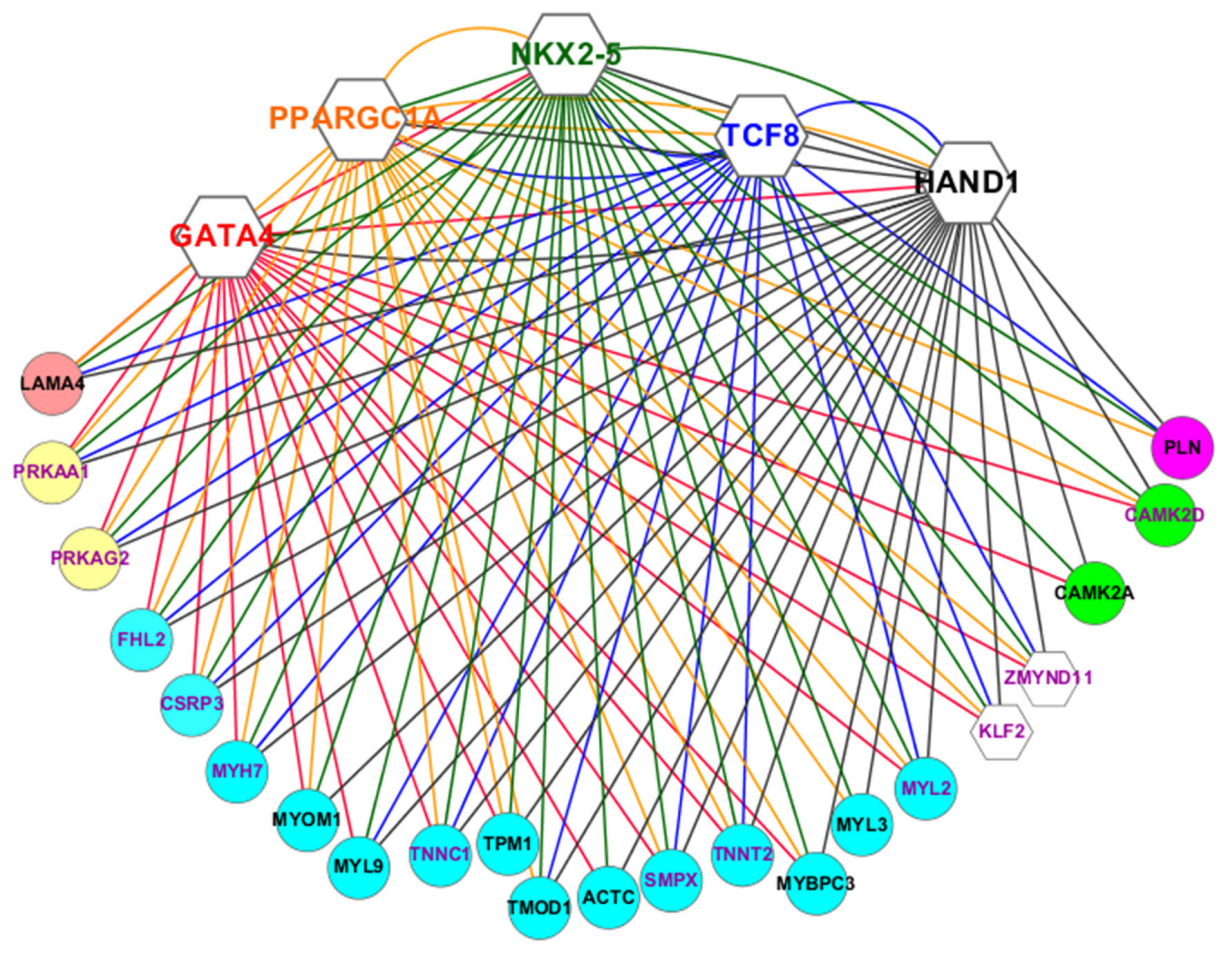
Co-expression network in CMs. The five transcription factors are shown as hexagons with gene symbols and their connections are shown by solid lines of the same colors e.g. GATA4 is in red and are connected to co-expressed genes by solid red lines. Genes are shown in circles and are color-coded to indicate their functional classifications. Red = regulation of contraction/adhesion, yellow = AMP kinases, blue = contractile genes, white = transcription factors, green = Ca/calmodulin kinases, purple = Ca handling protein. Gene labels in purple indicate genes that are co-expressed with all five transcription factors. Gene labels in black indicate genes that are co-expressed with three or four transcription factors.

We performed co-expression analysis to construct transcriptional networks of cardiac TFs and genes important for cardiac function [36]. First, we correlated the expression of cardiac TFs across the four groups of sample, hESCs, hESC-**V**CMs, hF-**V**CMs and hA-**V**CMs (Methods, Table S4) and found that a group of 4 TFs consisting of *HAND1, GATA4, NKX2-5* and *PPARGC1A* had significantly similar expression patterns and that their expression also correlated highly and significantly with a fifth TF, *TCF8*. *HAND1*, for instance, co-expressed with *GATA4, NKX2-5, PPARGC1A and TCF8* (Figure 4, Table S4). Likewise, *NKX2-5* also co-expressed with *GATA4, HAND1, PPARGC1A and TCF8*. We then showed that these 5 TFs co-expressed with many common genes, suggesting a co-regulatory relationship between these 5 TFs and other genes in the microarray. *HAND1, GATA4, NKX2-5, PPARGC1A* and *TCF8* co-expressed with 144, 133, 172, 160 and 112 genes respectively. These TFs also jointly co-expressed with many common genes. For example, among 71 genes that showed co-expression links with more than 5 of all 17 TFs, 39 co-expressed with *HAND1, GATA4, NKX2-5, PPARGC1A* and *TCF8*. In addition, 19 of these co-expressed exclusively with the 5 TFs, indicating that these TFs are major components of such a co-expressed gene network. Although we identified a relatively large number of co-expressed genes with *MEF2C* (108), *MESP1* (73) and *PPARA* (97), they did not form co-expression links with other TFs. The other 8 TFs co-expressed with far fewer genes (below 51). 

The gene ontology affiliations of genes which co-expressed with the 5 TFs were primarily associated with Ca^2+^ homeostasis, contraction, metabolism and transcriptional regulation. Totally, 39 genes had a co-expression relationship with all 5 TFs. Of these, seven (*MYH7, SMPX, TNNT2, MYL2, TNNC1, CSRP3* and *FHL2*) are involved in contraction/cytoskeleton (Figure 4). The rest included TFs (*KLF2* and *ZMYND11*), and regulators of the AMPK pathway *PRKAA1* and *PRKAG2*, etc. 88 genes correlated with at least 4 of the 5 TFs, including themselves *HAND1* and *NKX2-5* (Table S4). These analyses were then extended to develop a co-expression network, that bioinformatically shows highly significant and conserved patterns of expression between TFs and putative target genes. The resulting co-expression network consisted of genes associated with transcriptional regulation, contraction, metabolism and calcium handling processes and consisted of 5 TFs and 22 putative target genes (Figure 4). 

We further defined the regulatory structure of our co-expression network by examining the promoter regions of all 27 genes for binding sites of 4 TFs (GATA4, HAND1, NKX2-5 and TCF8) (Table 4, Table S5 for details). PPARGC1A was excluded from analysis because it mostly functions as a co-factor and does not have defined binding sites. Our analysis identified many binding sites for the 4 TFs among the promoters of the 27 genes. GATA4, HAND1, NKX2-5 and TCF8 predicted binding sites were found in the promoter regions of 9, 10, 23, 22 genes respectively. Many genes contained binding sites for multiple TFs in their promoters. 22 out of 27 genes contained binding sites for 2 or more TFs in their promoters, consistent with a regulatory relationship between the TFs analysed and the other genes in the co-expression network. Additionally, the TFs themselves were predicted to regulate each other. For instance, HAND1 promoter contained binding sites for all 4 TFs (GATA4, HAND1, NKX2-5 and TCF8). This is consistent with the co-expression relationship between *HAND1* and *GATA4, NKX2-5, PPARGC1A and TCF8.*

**Table 4:** Promoter analysis. The number below each TF indicates the number of putative binding sites in the region 1000bp up- and 300bp down-stream of any given gene.

Gene	GATA4	HAND1	NKX2.5	TCF8
ACTC1			2	
CAMK2A			2	1
CAMK2D		1	2	1
CSRP3	1	1	5	1
FHL2	1		8	3
GATA4				
HAND1	1	1	1	1
KLF2			3	
LAMA4		1		1
MYBPC3			2	1
MYH7			2	1
MYL2			2	1
MYL3		2	4	2
MYL9		1		
MYOM1	1		4	1
NKX2-5			2	3
PLN	2		5	3
PPARGC1A	1		3	1
PRKAA1			2	1
PRKAG2		1	4	2
SMPX		2	2	1
TMOD1			3	1
TNNC1		1	2	2
TNNT2			1	
TPM1	1	1		1
ZEB1	1		1	2
ZMYND11	2		2	3

## Discussion

Here, we describe the transcriptome of hESC-**V**CMs and compare them with their in vivo counterparts to evaluate their molecular phenotype and developmental status and to identify regulatory mechanisms that might underlie these differences. Our PCA results show that hESC-**V**CMs are developmentally less advanced than hF-**V**CMs of 18-20 weeks. In support of the above assertion, we also show that the expression of genes important for CM function (e.g. contraction, metabolism and heart development) are low in hESC-**V**CMs. Similar results have been reported between non-purified/mixed lineage hESC-CMs and/or whole fetal heart, but it was unclear whether these results were due to contaminating fibroblasts in whole fetal heart and/or the presence of pacemaker/atrial/ventricular cells in hESC-CM cultures [14,17]. Here we showed that ventricular-specific hESC-CMs are less mature than hF-CMs of 18-20 weeks. Consistent with our findings, He et al. claim that hESC-derived beating outgrowths have properties of APs anticipated in embryonic heart before 7 weeks of development [2]. Our staging, however, differs from that of Cao et al [14] who stated that hESC-derived cultures were most similar to 20 weeks old hF-**V**CMs. Such differences could be attributed to the limited enrichment of hESC-CMs in their cultures, which consisted of only 40-45% CMs. Consistent with this, hierarchical clustering showed that their enriched hESC-CMs were grouped more closely with embryoid bodies (consisting of a mixed cell population) than hF-**V**CMs. We also uniquely show that hESC-**V**CMs expressed markers of, and had low conduction velocity consistent with immature chamber myocardium [23]. Two groups have previously measured the conduction velocity of non-purified beating hESC-CMs clusters [11,37]. However, the results were heterogeneous and the authors indicated that this may partly stem from the presence of non-myocytes within the cell network which may electrically couple with CMs and thereby slow conduction [37]. We show that purified hESC-**V**CMs indeed had a conduction velocity(5.0±1.4 cm/s) much slower than that of adult human heart [25].

16% of genes are differentially expressed between hESC-**V**CMs and hF-**V**CMs by more than 2-fold. Interestingly, this 16% difference accounts for a 49% difference among gene sets as examined by GSEA, partly because differential expression of the same genes can contribute to the enrichment of multiple gene sets (e.g., *CCNB1* was found in 20 gene sets upregulated in hF-**V**CMs). An examination of gene sets (defined by function) rather than individual genes gives a more comprehensive overview of transcriptional differences among hESC-**V**CMs and hF-**V**CMs. By applying this approach, we confirm expression changes in contractile, fatty acid metabolic genes using our ventricular-specific system. In addition, we uniquely show that hESC-VCMs expressed lower levels of cell cycle and ROS metabolic genes and higher levels of genes associated with migration. In the setting of acute myocardial infarction, ROS is implicated in tissue necrosis and reperfusion injury [38]. Therefore, mechanisms that promote ROS enzyme up-regulation would be important to promote cell survival in the context of cell therapy. We found that hESC-**V**CMs expressed significantly lower levels of genes involved in cell cycle progression and telomere maintenance while genes involved in cellular senescence and apoptosis are up-regulated. It should be noted that proliferation of hESC-CMs can be affected by culture conditions [39]. Migration-related genes, however, are up-regulated in hESC-**V**CMs. Embryonic heart development involves complex morphogenic progression to transform the linear tube to a four-chambered heart. Thus, the motile phenotype of hESC-**V**CMs may reflect that of early embryonic CMs and may mean better abilities to home and migrate to injured sites for transplantation therapy.

We and others have reported that hESC-CMs display immature Ca^2+^ transient properties [8] and exhibit other defects such as a negative force-frequency response [40] and a lack of positive inotropy upon β-adrenergic stimulation [41]. Here, we have identified molecules which are dramatically down-regulated in hESC-**V**CMs and which may underlie the above defects. CAMKII is critically involved in the regulation of Ca^2+^ homeostasis through phosphorylation of Ca^2+^ handling proteins such as PLN [42] and RyR [43] and also participates in force-frequency response [43]. Other down-regulated genes include *FXYD1* (phospholemman) and *SRL* (sarcalumenin), which can regulate Ca^2+^ transient properties and modulate adrenergic stimulation [44,45]. In addition, sarcalumenin was among the 200 most abundant genes in hF-**V**CMs and hA-**V**CMs and was 11-fold lower in hESC-**V**CMs. Our group has previously shown that over-expression of calsequestrin, a Ca^2+^-handling protein absent in hESC-**V**CMs, can facilitate CM maturation [46]. The genes identified here represent new possible targets for mechanism-based maturation strategies. 

One of the major applications of hESC-CM research is to transplant these in vitro generated CMs into infarcted heart to repair damaged myocardium. Laflamme et al has previously shown that hESC-CM survival is significantly lower when transplanted into infarcted rat heart compared to uninjured heart and that the addition of pro-survival factors is required to improve graft survival [47]. Understanding factors that regulate hESC-**V**CM survival under ischemic environment is therefore crucial for hESC-**V**CM transplantation therapy. Here, we show that hESC-**V**CMs express lower levels of cardioprotective molecules that regulate ROS metabolism, *I*_KATP_, *I*_KNa_ and *I*_KCa_ etc and are correspondingly more vulnerable to hypoxia/reoxygenation injury than hF-**V**CMs. We also confirmed by functional analysis that *I*_KATP_ was depleted in hESC-**V**CMs compared to adult CMs. Factors that upregulate these molecules would therefore be of benefit to the use of hESC-**V**CMs in regenerative medicine. Another potential application for hESC-CMs is to use these cells as in vitro test beds for detecting pro-arrhythmic and/or cardiotoxic drugs [48,49]. Our hESC-VCMs express reduced levels of important channels and Ca^2+^ handling genes eg KCNJ2 and PLN etc, consistent with previous publications [8,17,50]. On a multicellular level, the random arrangement of CX43 and low conduction velocity reported here are also in line with our recent paper showing that the conduction pattern of hESC-VCMs cultured on 2-dimensional surfaces are immature and isotropic [51]. Successful drug testing requires that hESC-**V**CMs exhibit electrophysiological and survival properties similar to adult CMs in vivo. Here we demonstrate that the expression and function of specific ion channels and cardioprotective molecules are perturbed in hESC-VCMs compared to their in vivo counterparts. Although hESC-CMs may still have advantages over current cell models such as primary canine or rabbit Purkinje fibers or cell lines ectopically expressing the hERG ion channel, we urge that results of tests involving hESC-CMs be treated with caution as we recently reviewed [52]. 

Embryonic heart development involves the coordinate action of many TFs. To unravel these complex actions, we employed bioinformatics co-expression and promoter analyses. Our analyses suggest that genes involved in transcriptional regulation, contraction, energy metabolism and calcium homeostasis may be co-regulated on a transcriptional level. Interestingly, many genes identified in our network are already related via post-translational regulation or protein interaction, for example, CAMKII phosphorylates PLN to regulate Ca^2+^ homeostasis, which in turn determines contractile activity by modulating troponin/tropomyosin interaction. The co-expression of these molecules suggests that they may be commonly regulated on the mRNA (as well as protein) level. Our promoter analysis further reveals that HAND1, GATA4, NKX2-5 and TCF8 binding sites are present in the promoters of the majority of genes in the co-expression network, suggesting a regulatory relationship between these TFs and their putative target genes. We speculate that the five TFs (HAND1, GATA4, NKX2-5, PPARGC1A and TCF8) may play a role in the regulation of diverse processes important for cardiac function, however, only four of these TFs are known to be critical to heart development or function. GATA4 regulates the expression of *MYH7*(βMHC) [53] and acts synergistically with NKX2.5 to activate downstream targets [31]. The importance of these TFs is underscored by transgenic studies, which shows that null deletions of *Gata4, Nkx2.5* or *Hand1* arrest cardiac development in vivo. PPARGC1A is a co-regulator of the PPAR pathway, and is important for metabolic activity [54]. The fifth member of the group, TCF8, has not previously been associated with heart development and function. Mice null for Tcf8 had no reported heart abnormality [55]. In summary, we uncovered a transcriptional hierarchy involving 5TFs and genes important for CM development and function, and we postulate these 5 TFs may be crucial for hESC-CM maturation. Consistent with this, perinatal loss of Nkx2-5 in mice results in reduced contractile and Ca^2+^-handling parameters which are accompanied by decreased ion channel expression [56], and this is reminiscent of the contractile and electrophysiological defects of hESC-CMs. Agents that up-regulate these TFs may promote hESC-CM maturation in vitro.

## Conclusion

Differentiation of hESC into CMs can potentially represent an unlimited cell source for disease modeling and cell based therapies. However, caution should be taken to ensure their safety by comparing these in vitro generated CMs with in vivo standards. HESC-**V**CMs generated using current protocols are functionally immature and are vulnerable to injury. Mechanism-based in vitro maturation strategies would be crucial to facilitate the translation of hESC-CMs into clinical applications. 

## Supporting Information

Table S1 **Sample information.**
(DOCX)

Table S2 **The top 200 most abundant genes in hESC-VCMs, hF-VCMs and hA-VCMs.** Fold changes relative to hESC are shown.
(XLSX)

Table S3 **GSEA results showing all gene sets which displayed A) decreased and B) increased expression in hESC-VCMs relative to hF-VCMs.** FDR indicates the false discovery date and FDR<0.05 was considered significant. Size = number of genes present within gene sets.
(DOCX)

Table S4 **Genes co-expressed with 17 TFs.** ‘17TF-merged’ indicates the number of TFs that co-expressed with any particular gene. ‘5TF-merged’ shows the number of TFs from within the core 5 TFs (ie GATA4, HAND1, NKX2.5, PPARGC1A and TCF8, highlighted in bold) that co-expressed with any particular gene. ‘% core TF’ is the proportion of TFs (out of the total 17 TFs) that belong to the core cluster of 5 TFs. For instance, LAMA4 co-expressed with 8 TFs, ie ‘17TF-merged’ is 8. It co-expressed with all 5 of the core TFs (GATA4, HAND1, NKX2.5, PPARGC1A and TCF8) ie ‘5TF-merged’ is 5. ‘% core TF’ is 5 out of 8 ie 63%. Only genes that co-expressed with 4 or 5 of the 5 core TFs are shown.
(DOCX)

Table S5 **Promoter analysis of GATA4, NKX2.5, HAND1 and TCF8.**
(XLSX)

Figure S1 **QPCR analysis of selected genes in hESC-VCMs and hF-VCMs.** Expression was normalized to GAPDH. * p<0.05.
(TIF)

Methods S1 **Supporting methods.**
(DOCX)
