# Supplementary material for: Transcriptome-Guided Functional Analyses Reveal Novel Biological Properties and Regulatory Hierarchy of Human Embryonic Stem Cell-Derived Ventricular Cardiomyocytes Crucial for Maturation
Source: PLoS One. 2013 Oct 21;8(10):e77784. doi: 10.1371/journal.pone.0077784 (PMC3804624; doi:10.1371/journal.pone.0077784)
Supplement: Table S1 — Sample information. (DOCX) [file pone.0077784.s001.docx]

**Table S1 Sample information**

| Sample | Details |
| --- | --- |
| hESC | Undifferentiated HES2 cells, female |
| hESC-**V**CM | MLC2v-mcherry positive CM differentiated from HES2 cells |
| hF-**V**CM | 18-20 weeks |
| hA-**V**CM | Age 53-70 |
