# Supplementary material for: Transcriptome-Guided Functional Analyses Reveal Novel Biological Properties and Regulatory Hierarchy of Human Embryonic Stem Cell-Derived Ventricular Cardiomyocytes Crucial for Maturation
Source: PLoS One. 2013 Oct 21;8(10):e77784. doi: 10.1371/journal.pone.0077784 (PMC3804624; doi:10.1371/journal.pone.0077784)
Supplement: Table S3 — GSEA results showing all gene sets which displayed A) decreased and B) increased expression in hESC-VCMs relative to hF-VCMs. FDR indicates the false discovery date and FDR<0.05 was considered significant. Size = number of genes present within gene sets. (DOCX) [file pone.0077784.s003.docx]

**Table S3 GSEA results showing all gene sets which displayed A) decreased and B) increased expression in hESC-VCMs relative to hF-VCMs.** FDR indicates the false discovery date and FDR<0.05 was considered significant. Size = number of genes present within gene sets.

A

| NAME | SIZE | FDR q-val |
| --- | --- | --- |
| MITOTIC_PROMETAPHASE(GO-0000236) | 68 | 0 |
| M_PHASE_OF_MITOTIC_CELL_CYCLE(GO-0000087) | 133 | 0 |
| MITOSIS(GO-0007067) | 127 | 0 |
| CELLULAR_RESPIRATION(GO-0045333) | 105 | 0 |
| ACETYL-COA_METABOLIC_PROCESS(GO-0006084) | 25 | 0 |
| AEROBIC_RESPIRATION(GO-0009060) | 30 | 0 |
| STRIATED_MUSCLE_CONTRACTION(GO-0006941) | 27 | 0 |
| ELECTRON_TRANSPORT_CHAIN(GO-0022900) | 78 | 0 |
| RESPIRATORY_ELECTRON_TRANSPORT_CHAIN(GO-0022904) | 78 | 0 |
| REGULATION_OF_CHROMOSOME_SEGREGATION(GO-0051983) | 17 | 0 |
| TRICARBOXYLIC_ACID_CYCLE(GO-0006099) | 20 | 0 |
| MITOTIC_CELL_CYCLE(GO-0000278) | 389 | 0 |
| MUSCLE_CONTRACTION(GO-0006936) | 135 | 0 |
| DNA_STRAND_ELONGATION(GO-0022616) | 27 | 4.55E-05 |
| MUSCLE_FILAMENT_SLIDING(GO-0030049) | 34 | 1.66E-04 |
| DNA_STRAND_ELONGATION_INVOLVED_IN_DNA_REPLICATION(GO-0006271) | 25 | 1.94E-04 |
| ACTIN-MYOSIN_FILAMENT_SLIDING(GO-0033275) | 34 | 2.56E-04 |
| SPINDLE_CHECKPOINT(GO-0031577) | 32 | 3.82E-04 |
| CHROMOSOME_SEGREGATION(GO-0007059) | 61 | 3.62E-04 |
| DNA-DEPENDENT_DNA_REPLICATION(GO-0006261) | 46 | 3.75E-04 |
| MUSCLE_SYSTEM_PROCESS(GO-0003012) | 146 | 6.22E-04 |
| SISTER_CHROMATID_SEGREGATION(GO-0000819) | 25 | 7.37E-04 |
| MITOTIC_SISTER_CHROMATID_SEGREGATION(GO-0000070) | 24 | 0.001011 |
| MICROTUBULE_CYTOSKELETON_ORGANIZATION(GO-0000226) | 107 | 9.96E-04 |
| ACTIN_FILAMENT-BASED_MOVEMENT(GO-0030048) | 43 | 0.001587 |
| CELLULAR_RESPONSE_TO_REACTIVE_OXYGEN_SPECIES(GO-0034614) | 36 | 0.001599 |
| CELLULAR_RESPONSE_TO_HYDROGEN_PEROXIDE(GO-0070301) | 24 | 0.002145 |
| SPINDLE_ASSEMBLY_CHECKPOINT(GO-0071173) | 27 | 0.002114 |
| MITOTIC_CELL_CYCLE_SPINDLE_ASSEMBLY_CHECKPOINT(GO-0007094) | 26 | 0.00213 |
| MITOTIC_CELL_CYCLE_SPINDLE_CHECKPOINT(GO-0071174) | 29 | 0.002393 |
| MITOTIC_RECOMBINATION(GO-0006312) | 26 | 0.002517 |
| MICROTUBULE_ORGANIZING_CENTER_ORGANIZATION(GO-0031023) | 40 | 0.002497 |
| DNA_PACKAGING(GO-0006323) | 41 | 0.002632 |
| HEART_CONTRACTION(GO-0060047) | 15 | 0.003057 |
| NEGATIVE_REGULATION_OF_MITOSIS(GO-0045839) | 32 | 0.003255 |
| CARDIAC_MUSCLE_TISSUE_MORPHOGENESIS(GO-0055008) | 27 | 0.00332 |
| MUSCLE_ORGAN_MORPHOGENESIS(GO-0048644) | 29 | 0.003281 |
| TELOMERE_MAINTENANCE_VIA_SEMI-CONSERVATIVE_REPLICATION(GO-0032201) | 20 | 0.003228 |
| GENERATION_OF_PRECURSOR_METABOLITES_AND_ENERGY(GO-0006091) | 273 | 0.003339 |
| BRANCHED_CHAIN_FAMILY_AMINO_ACID_CATABOLIC_PROCESS(GO-0009083) | 17 | 0.003317 |
| DETERMINATION_OF_HEART_LEFT-RIGHT_ASYMMETRY(GO-0061371) | 24 | 0.003436 |
| CENTROSOME_ORGANIZATION(GO-0051297) | 35 | 0.003428 |
| CARBOXYLIC_ACID_CATABOLIC_PROCESS(GO-0046395) | 118 | 0.003757 |
| EMBRYONIC_HEART_TUBE_MORPHOGENESIS(GO-0003143) | 23 | 0.003899 |
| TELOMERE_MAINTENANCE_VIA_RECOMBINATION(GO-0000722) | 22 | 0.003813 |
| DNA_REPLICATION(GO-0006260) | 94 | 0.003975 |
| MICROTUBULE-BASED_PROCESS(GO-0007017) | 143 | 0.004116 |
| ENERGY_DERIVATION_BY_OXIDATION_OF_ORGANIC_COMPOUNDS(GO-0015980) | 225 | 0.004225 |
| MUSCLE_TISSUE_MORPHOGENESIS(GO-0060415) | 29 | 0.004293 |
| MITOTIC_SPINDLE_ORGANIZATION(GO-0007052) | 20 | 0.005069 |
| CENTROSOME_CYCLE(GO-0007098) | 23 | 0.005822 |
| STEROID_HORMONE_MEDIATED_SIGNALING_PATHWAY(GO-0043401) | 15 | 0.006183 |
| REGULATION_OF_CELL_CYCLE_PROCESS(GO-0010564) | 305 | 0.006386 |
| HYDROGEN_PEROXIDE_METABOLIC_PROCESS(GO-0042743) | 15 | 0.007155 |
| INTERPHASE_OF_MITOTIC_CELL_CYCLE(GO-0051329) | 258 | 0.008077 |
| POSITIVE_REGULATION_OF_CELL-SUBSTRATE_ADHESION(GO-0010811) | 18 | 0.008123 |
| VENTRICULAR_CARDIAC_MUSCLE_TISSUE_MORPHOGENESIS(GO-0055010) | 20 | 0.008159 |
| HEART_DEVELOPMENT(GO-0007507) | 148 | 0.009074 |
| HEART_LOOPING(GO-0001947) | 22 | 0.009037 |
| MUSCLE_FIBER_DEVELOPMENT(GO-0048747) | 18 | 0.009429 |
| RESPONSE_TO_HYDROGEN_PEROXIDE(GO-0042542) | 30 | 0.009565 |
| HISTONE_EXCHANGE(GO-0043486) | 17 | 0.009421 |
| CELLULAR_LIPID_CATABOLIC_PROCESS(GO-0044242) | 69 | 0.00985 |
| REGULATION_OF_MUSCLE_CONTRACTION(GO-0006937) | 41 | 0.009775 |
| INTERPHASE(GO-0051325) | 262 | 0.009856 |
| M-G1_TRANSITION_OF_MITOTIC_CELL_CYCLE(GO-0000216) | 70 | 0.011359 |
| FATTY_ACID_OXIDATION(GO-0019395) | 41 | 0.011698 |
| CHROMOSOME_ORGANIZATION(GO-0051276) | 316 | 0.012103 |
| ATP-DEPENDENT_CHROMATIN_REMODELING(GO-0043044) | 19 | 0.012084 |
| FATTY_ACID_CATABOLIC_PROCESS(GO-0009062) | 37 | 0.011955 |
| FATTY_ACID_TRANSPORT(GO-0015908) | 25 | 0.011938 |
| REGULATION_OF_STRIATED_MUSCLE_CONTRACTION(GO-0006942) | 20 | 0.012174 |
| ESTABLISHMENT_OF_CHROMOSOME_LOCALIZATION(GO-0051303) | 15 | 0.012016 |
| EMBRYONIC_HEART_TUBE_DEVELOPMENT(GO-0035050) | 26 | 0.012229 |
| FATTY_ACID_BETA-OXIDATION(GO-0006635) | 32 | 0.012613 |
| CELL_CYCLE_CHECKPOINT(GO-0000075) | 186 | 0.012498 |
| CYTOKINESIS(GO-0000910) | 58 | 0.013471 |
| REGULATION_OF_MITOTIC_METAPHASE-ANAPHASE_TRANSITION(GO-0030071) | 32 | 0.013346 |
| SPINDLE_ORGANIZATION(GO-0007051) | 34 | 0.013226 |
| REGULATION_OF_CELL_CYCLE_ARREST(GO-0071156) | 205 | 0.013169 |
| CARDIAC_MUSCLE_TISSUE_DEVELOPMENT(GO-0048738) | 47 | 0.013146 |
| DNA_CONFORMATION_CHANGE(GO-0071103) | 67 | 0.013937 |
| VASCULOGENESIS(GO-0001570) | 23 | 0.015183 |
| REGULATION_OF_MICROTUBULE-BASED_PROCESS(GO-0032886) | 56 | 0.015345 |
| PROTEIN-DNA_COMPLEX_ASSEMBLY(GO-0065004) | 30 | 0.015245 |
| MUSCLE_CELL_DEVELOPMENT(GO-0055001) | 39 | 0.015487 |
| DNA_RECOMBINATION(GO-0006310) | 81 | 0.016076 |
| RESPONSE_TO_REACTIVE_OXYGEN_SPECIES(GO-0000302) | 49 | 0.01667 |
| POSITIVE_REGULATION_OF_CELL-MATRIX_ADHESION(GO-0001954) | 16 | 0.016956 |
| HEART_MORPHOGENESIS(GO-0003007) | 84 | 0.01681 |
| NUCLEOTIDE-EXCISION_REPAIR__DNA_GAP_FILLING(GO-0006297) | 19 | 0.016729 |
| REGULATION_OF_MICROTUBULE_CYTOSKELETON_ORGANIZATION(GO-0070507) | 48 | 0.018741 |
| ACTIVATION_OF_PROTEIN_KINASE_A_ACTIVITY(GO-0034199) | 16 | 0.01873 |
| TRANSLATIONAL_ELONGATION(GO-0006414) | 91 | 0.019484 |
| MUSCLE_TISSUE_DEVELOPMENT(GO-0060537) | 87 | 0.019412 |
| CENTROSOME_DUPLICATION(GO-0051298) | 16 | 0.020953 |
| LIPID_OXIDATION(GO-0034440) | 42 | 0.022546 |
| REGULATION_OF_CYTOKINESIS(GO-0032465) | 19 | 0.02263 |
| LIPID_CATABOLIC_PROCESS(GO-0016042) | 84 | 0.022471 |
| LONG-CHAIN_FATTY_ACID_TRANSPORT(GO-0015909) | 21 | 0.022726 |
| CARDIAC_VENTRICLE_MORPHOGENESIS(GO-0003208) | 35 | 0.023027 |
| G1-S_TRANSITION_OF_MITOTIC_CELL_CYCLE(GO-0000082) | 132 | 0.027002 |
| ACTOMYOSIN_STRUCTURE_ORGANIZATION(GO-0031032) | 22 | 0.029589 |
| MUSCLE_ORGAN_DEVELOPMENT(GO-0007517) | 103 | 0.033745 |
| REACTIVE_OXYGEN_SPECIES_METABOLIC_PROCESS(GO-0072593) | 42 | 0.035551 |
| POSITIVE_REGULATION_OF_CELL_DIVISION(GO-0051781) | 19 | 0.036732 |
| REGULATION_OF_ATPASE_ACTIVITY(GO-0043462) | 21 | 0.037018 |
| CELL_DIVISION(GO-0051301) | 73 | 0.038581 |
| STRIATED_MUSCLE_CELL_DEVELOPMENT(GO-0055002) | 32 | 0.03848 |
| CARDIAC_MUSCLE_CELL_DIFFERENTIATION(GO-0055007) | 20 | 0.040211 |
| CHROMATIN_ASSEMBLY_OR_DISASSEMBLY(GO-0006333) | 43 | 0.039855 |
| S_PHASE_OF_MITOTIC_CELL_CYCLE(GO-0000084) | 97 | 0.042255 |
| VIRAL_TRANSCRIPTION(GO-0019083) | 81 | 0.042273 |
| DETERMINATION_OF_LEFT-RIGHT_SYMMETRY(GO-0007368) | 35 | 0.042718 |
| TRANSLATIONAL_TERMINATION(GO-0006415) | 83 | 0.045539 |
| PYRUVATE_METABOLIC_PROCESS(GO-0006090) | 21 | 0.045962 |
| DNA_METABOLIC_PROCESS(GO-0006259) | 396 | 0.04595 |
| CELLULAR_PROTEIN_COMPLEX_DISASSEMBLY(GO-0043624) | 92 | 0.046882 |
| OXIDATION-REDUCTION_PROCESS(GO-0055114) | 372 | 0.048087 |

B

| NAME | SIZE | | FDR q-val |
| --- | --- | --- | --- |
| CELLULAR_RESPONSE_TO_TYPE_I_INTERFERON(GO-0071357) | | 62 | 0 |
| RESPONSE_TO_TYPE_I_INTERFERON(GO-0034340) | | 64 | 0 |
| TYPE_I_INTERFERON-MEDIATED_SIGNALING_PATHWAY(GO-0060337) | | 62 | 0 |
| RESPONSE_TO_CYTOKINE_STIMULUS(GO-0034097) | | 314 | 0 |
| CELLULAR_RESPONSE_TO_CYTOKINE_STIMULUS(GO-0071345) | | 272 | 0 |
| CYTOKINE-MEDIATED_SIGNALING_PATHWAY(GO-0019221) | | 232 | 0 |
| INNATE_IMMUNE_RESPONSE(GO-0045087) | | 324 | 0 |
| RESPONSE_TO_ENDOPLASMIC_RETICULUM_STRESS(GO-0034976) | | 80 | 0 |
| REGULATION_OF_RESPONSE_TO_STRESS(GO-0080134) | | 446 | 0 |
| REGULATION_OF_CYTOKINE_PRODUCTION(GO-0001817) | | 226 | 0 |
| RESPONSE_TO_INTERFERON-GAMMA(GO-0034341) | | 88 | 0 |
| ENDOPLASMIC_RETICULUM_UNFOLDED_PROTEIN_RESPONSE(GO-0030968) | | 71 | 0 |
| REGULATION_OF_I-KAPPAB_KINASE-NF-KAPPAB_CASCADE(GO-0043122) | | 132 | 0 |
| RESPONSE_TO_BIOTIC_STIMULUS(GO-0009607) | | 206 | 0 |
| ACTIVATION_OF_SIGNALING_PROTEIN_ACTIVITY_INVOLVED_IN_UNFOLDED_PROTEIN_RESPONSE(GO-0006987) | | 61 | 0 |
| REGULATION_OF_WOUND_HEALING(GO-0061041) | | 53 | 0 |
| CELLULAR_RESPONSE_TO_TOPOLOGICALLY_INCORRECT_PROTEIN(GO-0035967) | | 76 | 0 |
| REGULATION_OF_CELL_MIGRATION(GO-0030334) | | 237 | 0 |
| NEGATIVE_REGULATION_OF_SIGNAL_TRANSDUCTION(GO-0009968) | | 334 | 0 |
| NEGATIVE_REGULATION_OF_IMMUNE_SYSTEM_PROCESS(GO-0002683) | | 81 | 0 |
| REGULATION_OF_CYTOKINE_BIOSYNTHETIC_PROCESS(GO-0042035) | | 52 | 0 |
| REGULATION_OF_BLOOD_COAGULATION(GO-0030193) | | 42 | 0 |
| POSITIVE_REGULATION_OF_LOCOMOTION(GO-0040017) | | 152 | 0 |
| REGULATION_OF_CYTOKINE-MEDIATED_SIGNALING_PATHWAY(GO-0001959) | | 59 | 0 |
| CELLULAR_RESPONSE_TO_INTERFERON-GAMMA(GO-0071346) | | 76 | 0 |
| POSITIVE_REGULATION_OF_I-KAPPAB_KINASE-NF-KAPPAB_CASCADE(GO-0043123) | | 113 | 0 |
| REGULATION_OF_CHEMOTAXIS(GO-0050920) | | 62 | 0 |
| RESPONSE_TO_VIRUS(GO-0009615) | | 98 | 0 |
| SKELETAL_SYSTEM_DEVELOPMENT(GO-0001501) | | 155 | 0 |
| POSITIVE_REGULATION_OF_CELLULAR_COMPONENT_MOVEMENT(GO-0051272) | | 154 | 0 |
| POSITIVE_REGULATION_OF_CELL_MOTILITY(GO-2000147) | | 148 | 0 |
| POSITIVE_REGULATION_OF_CELL_MIGRATION(GO-0030335) | | 145 | 0 |
| RESPONSE_TO_MOLECULE_OF_BACTERIAL_ORIGIN(GO-0002237) | | 69 | 0 |
| POSITIVE_REGULATION_OF_CYTOKINE_BIOSYNTHETIC_PROCESS(GO-0042108) | | 34 | 0 |
| REGULATION_OF_INTERLEUKIN-6_PRODUCTION(GO-0032675) | | 37 | 0 |
| ACTIVATION_OF_PRO-APOPTOTIC_GENE_PRODUCTS(GO-0008633) | | 30 | 0 |
| CELLULAR_RESPONSE_TO_MOLECULE_OF_BACTERIAL_ORIGIN(GO-0071219) | | 38 | 0 |
| REGULATION_OF_LOCOMOTION(GO-0040012) | | 267 | 0 |
| RESPONSE_TO_UNFOLDED_PROTEIN(GO-0006986) | | 90 | 0 |
| POSITIVE_REGULATION_OF_BEHAVIOR(GO-0048520) | | 52 | 0 |
| OLIGOSACCHARIDE_BIOSYNTHETIC_PROCESS(GO-0009312) | | 35 | 0 |
| NEGATIVE_REGULATION_OF_PEPTIDASE_ACTIVITY(GO-0010466) | | 76 | 0 |
| POSITIVE_REGULATION_OF_CHEMOTAXIS(GO-0050921) | | 47 | 0 |
| REGULATION_OF_CELLULAR_COMPONENT_MOVEMENT(GO-0051270) | | 266 | 0 |
| POSITIVE_REGULATION_OF_NUCLEASE_ACTIVITY(GO-0032075) | | 63 | 2.57E-05 |
| NEGATIVE_REGULATION_OF_ENDOPEPTIDASE_ACTIVITY(GO-0010951) | | 73 | 2.62E-05 |
| RESPONSE_TO_BACTERIUM(GO-0009617) | | 103 | 2.68E-05 |
| POSITIVE_REGULATION_OF_REACTIVE_OXYGEN_SPECIES_METABOLIC_PROCESS(GO-2000379) | | 17 | 2.74E-05 |
| REGULATION_OF_CELL_MOTILITY(GO-2000145) | | 254 | 2.80E-05 |
| POSITIVE_REGULATION_OF_LEUKOCYTE_CHEMOTAXIS(GO-0002690) | | 37 | 2.86E-05 |
| INFLAMMATORY_RESPONSE(GO-0006954) | | 137 | 6.89E-05 |
| POSITIVE_REGULATION_OF_LEUKOCYTE_MIGRATION(GO-0002687) | | 41 | 7.01E-05 |
| POSITIVE_REGULATION_OF_CYTOKINE_PRODUCTION(GO-0001819) | | 116 | 7.14E-05 |
| NEGATIVE_REGULATION_OF_HYDROLASE_ACTIVITY(GO-0051346) | | 136 | 7.27E-05 |
| REGULATION_OF_ENDOPEPTIDASE_ACTIVITY(GO-0052548) | | 156 | 7.41E-05 |
| CELLULAR_RESPONSE_TO_LIPOPOLYSACCHARIDE(GO-0071222) | | 34 | 7.55E-05 |
| EPIDERMIS_DEVELOPMENT(GO-0008544) | | 93 | 7.70E-05 |
| INTERFERON-GAMMA-MEDIATED_SIGNALING_PATHWAY(GO-0060333) | | 68 | 8.95E-05 |
| ANTI-APOPTOSIS(GO-0006916) | | 151 | 1.49E-04 |
| POSITIVE_REGULATION_OF_SIGNAL_TRANSDUCTION(GO-0009967) | | 452 | 1.49E-04 |
| REGULATION_OF_PROTEIN_SECRETION(GO-0050708) | | 73 | 1.51E-04 |
| POSITIVE_REGULATION_OF_STRESS-ACTIVATED_PROTEIN_KINASE_SIGNALING_CASCADE(GO-0070304) | | 33 | 1.51E-04 |
| NEGATIVE_REGULATION_OF_CYTOKINE_PRODUCTION(GO-0001818) | | 80 | 1.53E-04 |
| DOLICHOL-LINKED_OLIGOSACCHARIDE_BIOSYNTHETIC_PROCESS(GO-0006488) | | 29 | 1.54E-04 |
| CELL_MOTILITY(GO-0048870) | | 343 | 1.55E-04 |
| REGULATION_OF_PEPTIDASE_ACTIVITY(GO-0052547) | | 163 | 1.58E-04 |
| NEGATIVE_REGULATION_OF_APOPTOTIC_PROCESS(GO-0043066) | | 336 | 1.60E-04 |
| REGULATION_OF_BEHAVIOR(GO-0050795) | | 77 | 1.62E-04 |
| NEGATIVE_REGULATION_OF_TYPE_I_INTERFERON_PRODUCTION(GO-0032480) | | 27 | 1.65E-04 |
| NEGATIVE_REGULATION_OF_PROGRAMMED_CELL_DEATH(GO-0043069) | | 337 | 1.68E-04 |
| RESPONSE_TO_LIPOPOLYSACCHARIDE(GO-0032496) | | 59 | 1.84E-04 |
| REGULATION_OF_INTRACELLULAR_PROTEIN_KINASE_CASCADE(GO-0010627) | | 377 | 2.19E-04 |
| NEGATIVE_REGULATION_OF_IMMUNE_RESPONSE(GO-0050777) | | 25 | 2.28E-04 |
| NEUTRAL_AMINO_ACID_TRANSPORT(GO-0015804) | | 16 | 2.31E-04 |
| NEGATIVE_REGULATION_OF_COAGULATION(GO-0050819) | | 28 | 2.34E-04 |
| REGULATION_OF_INNATE_IMMUNE_RESPONSE(GO-0045088) | | 166 | 2.78E-04 |
| RESPONSE_TO_INTERLEUKIN-1(GO-0070555) | | 38 | 2.93E-04 |
| REGULATION_OF_INFLAMMATORY_RESPONSE(GO-0050727) | | 63 | 2.97E-04 |
| POSITIVE_REGULATION_OF_PROTEIN_TRANSPORT(GO-0051222) | | 92 | 3.01E-04 |
| POSITIVE_REGULATION_OF_NF-KAPPAB_TRANSCRIPTION_FACTOR_ACTIVITY(GO-0051092) | | 82 | 3.05E-04 |
| CELL_MIGRATION(GO-0016477) | | 319 | 3.09E-04 |
| LEUKOCYTE_MIGRATION(GO-0050900) | | 152 | 3.48E-04 |
| NEGATIVE_REGULATION_OF_BLOOD_COAGULATION(GO-0030195) | | 26 | 3.53E-04 |
| CHEMOTAXIS(GO-0006935) | | 380 | 3.60E-04 |
| VESICLE_TARGETING__TO__FROM_OR_WITHIN_GOLGI(GO-0048199) | | 20 | 3.87E-04 |
| POSITIVE_REGULATION_OF_INTRACELLULAR_PROTEIN_KINASE_CASCADE(GO-0010740) | | 260 | 4.35E-04 |
| POSITIVE_REGULATION_OF_TRANSCRIPTION_FACTOR_IMPORT_INTO_NUCLEUS(GO-0042993) | | 21 | 4.40E-04 |
| NEGATIVE_REGULATION_OF_CELL_DEATH(GO-0060548) | | 345 | 4.45E-04 |
| LIPOPOLYSACCHARIDE-MEDIATED_SIGNALING_PATHWAY(GO-0031663) | | 17 | 4.69E-04 |
| REGULATION_OF_CELL_ADHESION_MEDIATED_BY_INTEGRIN(GO-0033628) | | 24 | 4.75E-04 |
| EXTRACELLULAR_MATRIX_ORGANIZATION(GO-0030198) | | 40 | 5.50E-04 |
| CELL_MORPHOGENESIS_INVOLVED_IN_DIFFERENTIATION(GO-0000904) | | 356 | 5.72E-04 |
| TRANSFERRIN_TRANSPORT(GO-0033572) | | 29 | 6.09E-04 |
| POSITIVE_REGULATION_OF_SEQUENCE-SPECIFIC_DNA_BINDING_TRANSCRIPTION_FACTOR_ACTIVITY(GO-0051091) | | 135 | 6.38E-04 |
| EXTRACELLULAR_STRUCTURE_ORGANIZATION(GO-0043062) | | 40 | 6.45E-04 |
| REGULATION_OF_CELL_PROJECTION_ASSEMBLY(GO-0060491) | | 28 | 6.45E-04 |
| POSITIVE_REGULATION_OF_SECRETION(GO-0051047) | | 102 | 6.52E-04 |
| POSITIVE_REGULATION_OF_STRESS-ACTIVATED_MAPK_CASCADE(GO-0032874) | | 32 | 6.59E-04 |
| RESPONSE_TO_DSRNA(GO-0043331) | | 16 | 7.85E-04 |
| POSITIVE_REGULATION_OF_INFLAMMATORY_RESPONSE(GO-0050729) | | 26 | 8.04E-04 |
| POSITIVE_REGULATION_OF_OXIDOREDUCTASE_ACTIVITY(GO-0051353) | | 26 | 8.76E-04 |
| POSITIVE_REGULATION_OF_JNK_CASCADE(GO-0046330) | | 28 | 8.85E-04 |
| REGULATION_OF_CELL-CELL_ADHESION(GO-0022407) | | 45 | 8.98E-04 |
| REGULATION_OF_VESICLE-MEDIATED_TRANSPORT(GO-0060627) | | 114 | 9.06E-04 |
| REGULATION_OF_PROTEOLYSIS(GO-0030162) | | 85 | 9.52E-04 |
| POSITIVE_REGULATION_OF_PROTEIN_IMPORT_INTO_NUCLEUS(GO-0042307) | | 38 | 9.70E-04 |
| REGULATION_OF_JNK_CASCADE(GO-0046328) | | 75 | 9.77E-04 |
| REGULATION_OF_SEQUENCE-SPECIFIC_DNA_BINDING_TRANSCRIPTION_FACTOR_ACTIVITY(GO-0051090) | | 218 | 9.79E-04 |
| POSITIVE_REGULATION_OF_NUCLEOCYTOPLASMIC_TRANSPORT(GO-0046824) | | 44 | 9.86E-04 |
| POSITIVE_REGULATION_OF_ENDOCYTOSIS(GO-0045807) | | 34 | 0.001004 |
| BONE_DEVELOPMENT(GO-0060348) | | 34 | 0.001007 |
| NEGATIVE_REGULATION_OF_CELL_ADHESION(GO-0007162) | | 51 | 0.00101 |
| REGULATION_OF_VASCULAR_ENDOTHELIAL_GROWTH_FACTOR_PRODUCTION(GO-0010574) | | 16 | 0.001015 |
| DEFENSE_RESPONSE_TO_VIRUS(GO-0051607) | | 29 | 0.001024 |
| REGULATION_OF_CELL_PROJECTION_ORGANIZATION(GO-0031344) | | 111 | 0.001029 |
| PEPTIDE_TRANSPORT(GO-0015833) | | 18 | 0.001032 |
| NEGATIVE_REGULATION_OF_VIRAL_REPRODUCTION(GO-0048525) | | 17 | 0.001057 |
| NEGATIVE_REGULATION_OF_CELL_MIGRATION(GO-0030336) | | 71 | 0.00107 |
| CELL_MORPHOGENESIS(GO-0000902) | | 417 | 0.001098 |
| NEGATIVE_REGULATION_OF_RESPONSE_TO_CYTOKINE_STIMULUS(GO-0060761) | | 17 | 0.001106 |
| REGULATION_OF_SECRETION(GO-0051046) | | 227 | 0.001108 |
| ACTIVATION_OF_INNATE_IMMUNE_RESPONSE(GO-0002218) | | 92 | 0.001121 |
| NEGATIVE_REGULATION_OF_CELLULAR_COMPONENT_MOVEMENT(GO-0051271) | | 78 | 0.001188 |
| REGULATION_OF_PROTEIN_TRANSPORT(GO-0051223) | | 156 | 0.001254 |
| RESPONSE_TO_GLUCOSE_STIMULUS(GO-0009749) | | 26 | 0.001254 |
| GOLGI_VESICLE_TRANSPORT(GO-0048193) | | 102 | 0.001264 |
| CELL_ACTIVATION(GO-0001775) | | 302 | 0.001274 |
| REGULATION_OF_ENDOCYTOSIS(GO-0030100) | | 70 | 0.001295 |
| REGULATION_OF_MAP_KINASE_ACTIVITY(GO-0043405) | | 151 | 0.001316 |
| SENSORY_ORGAN_DEVELOPMENT(GO-0007423) | | 109 | 0.001326 |
| POSITIVE_REGULATION_OF_NITRIC_OXIDE_BIOSYNTHETIC_PROCESS(GO-0045429) | | 15 | 0.001326 |
| LEUKOCYTE_ACTIVATION(GO-0045321) | | 119 | 0.001335 |
| INNATE_IMMUNE_RESPONSE-ACTIVATING_SIGNAL_TRANSDUCTION(GO-0002758) | | 91 | 0.001355 |
| NEGATIVE_REGULATION_OF_VIRAL_GENOME_REPLICATION(GO-0045071) | | 17 | 0.001365 |
| NEGATIVE_REGULATION_OF_CELL_MOTILITY(GO-2000146) | | 73 | 0.001384 |
| GLYCOPROTEIN_METABOLIC_PROCESS(GO-0009100) | | 195 | 0.001403 |
| REGULATION_OF_CELL_MORPHOGENESIS(GO-0022604) | | 152 | 0.00145 |
| DIGESTIVE_TRACT_DEVELOPMENT(GO-0048565) | | 34 | 0.001526 |
| CELL_AGING(GO-0007569) | | 33 | 0.001528 |
| POSITIVE_REGULATION_OF_INTERLEUKIN-8_PRODUCTION(GO-0032757) | | 16 | 0.001539 |
| REGULATION_OF_DEFENSE_RESPONSE_TO_VIRUS(GO-0050688) | | 47 | 0.001543 |
| POSITIVE_REGULATION_OF_INNATE_IMMUNE_RESPONSE(GO-0045089) | | 112 | 0.00155 |
| REGULATION_OF_STRESS-ACTIVATED_MAPK_CASCADE(GO-0032872) | | 87 | 0.001554 |
| POST-TRANSLATIONAL_PROTEIN_MODIFICATION(GO-0043687) | | 160 | 0.001561 |
| PATTERN_RECOGNITION_RECEPTOR_SIGNALING_PATHWAY(GO-0002221) | | 88 | 0.001563 |
| NEGATIVE_REGULATION_OF_ENDOTHELIAL_CELL_PROLIFERATION(GO-0001937) | | 18 | 0.001569 |
| NEGATIVE_REGULATION_OF_PROTEIN_KINASE_ACTIVITY(GO-0006469) | | 91 | 0.001618 |
| RESPONSE_TO_NUTRIENT(GO-0007584) | | 71 | 0.001629 |
| CELLULAR_RESPONSE_TO_MECHANICAL_STIMULUS(GO-0071260) | | 43 | 0.001649 |
| NEGATIVE_REGULATION_OF_CELL_DIFFERENTIATION(GO-0045596) | | 179 | 0.00166 |
| SECRETION_BY_CELL(GO-0032940) | | 197 | 0.001751 |
| REGULATION_OF_INTERFERON-GAMMA_PRODUCTION(GO-0032649) | | 34 | 0.001751 |
| NEGATIVE_REGULATION_OF_CELL-CELL_ADHESION(GO-0022408) | | 18 | 0.001762 |
| MORPHOGENESIS_OF_A_BRANCHING_EPITHELIUM(GO-0061138) | | 31 | 0.001763 |
| REGULATION_OF_MAPK_CASCADE(GO-0043408) | | 191 | 0.001765 |
| RESPONSE_TO_EXTRACELLULAR_STIMULUS(GO-0009991) | | 123 | 0.001774 |
| REGULATION_OF_ACTIN_POLYMERIZATION_OR_DEPOLYMERIZATION(GO-0008064) | | 32 | 0.001799 |
| ANTERIOR-POSTERIOR_PATTERN_SPECIFICATION(GO-0009952) | | 26 | 0.00181 |
| CELLULAR_RESPONSE_TO_EXTRACELLULAR_STIMULUS(GO-0031668) | | 81 | 0.001845 |
| AXON_GUIDANCE(GO-0007411) | | 270 | 0.001865 |
| POSITIVE_REGULATION_OF_INTRACELLULAR_PROTEIN_TRANSPORT(GO-0090316) | | 46 | 0.001907 |
| POSITIVE_REGULATION_OF_EXOCYTOSIS(GO-0045921) | | 19 | 0.001911 |
| MORPHOGENESIS_OF_A_BRANCHING_STRUCTURE(GO-0001763) | | 41 | 0.001981 |
| REGULATION_OF_REACTIVE_OXYGEN_SPECIES_METABOLIC_PROCESS(GO-2000377) | | 34 | 0.001985 |
| REGULATION_OF_INTERLEUKIN-12_PRODUCTION(GO-0032655) | | 23 | 0.001992 |
| REGULATION_OF_PROTEIN_CATABOLIC_PROCESS(GO-0042176) | | 87 | 0.001993 |
| NEGATIVE_REGULATION_OF_KINASE_ACTIVITY(GO-0033673) | | 98 | 0.001996 |
| POSITIVE_REGULATION_OF_MONONUCLEAR_CELL_PROLIFERATION(GO-0032946) | | 51 | 0.001997 |
| RESPONSE_TO_TUMOR_NECROSIS_FACTOR(GO-0034612) | | 53 | 0.002 |
| REGULATION_OF_CELL_DIFFERENTIATION(GO-0045595) | | 442 | 0.002005 |
| REGULATION_OF_T_CELL_PROLIFERATION(GO-0042129) | | 47 | 0.002165 |
| TOLL-LIKE_RECEPTOR_SIGNALING_PATHWAY(GO-0002224) | | 79 | 0.00217 |
| AXONOGENESIS(GO-0007409) | | 302 | 0.00228 |
| OLIGOSACCHARIDE_METABOLIC_PROCESS(GO-0009311) | | 49 | 0.002282 |
| EMBRYONIC_DIGESTIVE_TRACT_DEVELOPMENT(GO-0048566) | | 17 | 0.002361 |
| ANTIGEN_PROCESSING_AND_PRESENTATION_OF_PEPTIDE_ANTIGEN(GO-0048002) | | 89 | 0.002379 |
| LEUKOCYTE_ACTIVATION_INVOLVED_IN_IMMUNE_RESPONSE(GO-0002366) | | 31 | 0.002393 |
| AGING(GO-0007568) | | 40 | 0.002521 |
| LIPOPROTEIN_METABOLIC_PROCESS(GO-0042157) | | 56 | 0.002566 |
| NEURON_PROJECTION_MORPHOGENESIS(GO-0048812) | | 316 | 0.002624 |
| REGULATION_OF_BLOOD_VESSEL_ENDOTHELIAL_CELL_MIGRATION(GO-0043535) | | 27 | 0.002654 |
| SECRETION(GO-0046903) | | 222 | 0.002676 |
| AMINO_ACID_TRANSPORT(GO-0006865) | | 72 | 0.002762 |
| NEURON_PROJECTION_DEVELOPMENT(GO-0031175) | | 340 | 0.002781 |
| REGULATION_OF_KINASE_ACTIVITY(GO-0043549) | | 427 | 0.002789 |
| INDUCTION_OF_APOPTOSIS(GO-0006917) | | 267 | 0.002822 |
| REGULATION_OF_CELL_ADHESION(GO-0030155) | | 140 | 0.002828 |
| CARBOHYDRATE_BIOSYNTHETIC_PROCESS(GO-0016051) | | 116 | 0.002841 |
| REGULATION_OF_PROTEIN_KINASE_ACTIVITY(GO-0045859) | | 408 | 0.002847 |
| POSITIVE_REGULATION_OF_INTRACELLULAR_TRANSPORT(GO-0032388) | | 51 | 0.00286 |
| CELLULAR_RESPONSE_TO_INTERLEUKIN-1(GO-0071347) | | 25 | 0.003038 |
| CELL_CHEMOTAXIS(GO-0060326) | | 72 | 0.003063 |
| REGULATION_OF_SMOOTH_MUSCLE_CELL_MIGRATION(GO-0014910) | | 17 | 0.003147 |
| REGULATION_OF_TOLL-LIKE_RECEPTOR_SIGNALING_PATHWAY(GO-0034121) | | 18 | 0.003163 |
| POSITIVE_REGULATION_OF_PROGRAMMED_CELL_DEATH(GO-0043068) | | 369 | 0.003239 |
| POSITIVE_REGULATION_OF_PROTEIN_SECRETION(GO-0050714) | | 51 | 0.003242 |
| NEGATIVE_REGULATION_OF_CATALYTIC_ACTIVITY(GO-0043086) | | 370 | 0.003252 |
| POSITIVE_REGULATION_OF_T_CELL_PROLIFERATION(GO-0042102) | | 34 | 0.003295 |
| DEVELOPMENT_OF_PRIMARY_SEXUAL_CHARACTERISTICS(GO-0045137) | | 59 | 0.003345 |
| BRANCHING_MORPHOGENESIS_OF_A_TUBE(GO-0048754) | | 35 | 0.003355 |
| NEGATIVE_REGULATION_OF_LIPID_TRANSPORT(GO-0032369) | | 17 | 0.003371 |
| NEGATIVE_REGULATION_OF_TRANSFERASE_ACTIVITY(GO-0051348) | | 106 | 0.003433 |
| PROTEIN_KINASE_B_SIGNALING_CASCADE(GO-0043491) | | 15 | 0.003455 |
| REGULATION_OF_CYTOKINE_PRODUCTION_INVOLVED_IN_IMMUNE_RESPONSE(GO-0002718) | | 26 | 0.003484 |
| POSITIVE_REGULATION_OF_APOPTOTIC_PROCESS(GO-0043065) | | 367 | 0.003551 |
| EAR_DEVELOPMENT(GO-0043583) | | 35 | 0.003617 |
| URETERIC_BUD_MORPHOGENESIS(GO-0060675) | | 19 | 0.003709 |
| MESENCHYME_DEVELOPMENT(GO-0060485) | | 56 | 0.003799 |
| POSITIVE_REGULATION_OF_LYMPHOCYTE_PROLIFERATION(GO-0050671) | | 50 | 0.003806 |
| ANTIGEN_PROCESSING_AND_PRESENTATION_OF_PEPTIDE_ANTIGEN_VIA_MHC_CLASS_I(GO-0002474) | | 86 | 0.003902 |
| ADHERENS_JUNCTION_ORGANIZATION(GO-0034332) | | 35 | 0.00392 |
| CELLULAR_RESPONSE_TO_NUTRIENT_LEVELS(GO-0031669) | | 77 | 0.004069 |
| T_CELL_ACTIVATION(GO-0042110) | | 60 | 0.004069 |
| NEGATIVE_REGULATION_OF_INTRACELLULAR_PROTEIN_KINASE_CASCADE(GO-0010741) | | 68 | 0.004082 |
| URETERIC_BUD_DEVELOPMENT(GO-0001657) | | 27 | 0.004216 |
| POSITIVE_REGULATION_OF_CELL_DEVELOPMENT(GO-0010720) | | 71 | 0.004219 |
| NERVE_DEVELOPMENT(GO-0021675) | | 15 | 0.00422 |
| CELLULAR_MEMBRANE_ORGANIZATION(GO-0016044) | | 309 | 0.00423 |
| RESPONSE_TO_MECHANICAL_STIMULUS(GO-0009612) | | 55 | 0.004234 |
| REGULATION_OF_EPITHELIAL_TO_MESENCHYMAL_TRANSITION(GO-0010717) | | 31 | 0.004371 |
| IMMUNE_RESPONSE-REGULATING_SIGNALING_PATHWAY(GO-0002764) | | 175 | 0.004385 |
| NEGATIVE_REGULATION_OF_SEQUENCE-SPECIFIC_DNA_BINDING_TRANSCRIPTION_FACTOR_ACTIVITY(GO-0043433) | | 79 | 0.004399 |
| LIPID_BIOSYNTHETIC_PROCESS(GO-0008610) | | 242 | 0.00445 |
| NEURON_DEVELOPMENT(GO-0048666) | | 370 | 0.004458 |
| NEGATIVE_REGULATION_OF_EPITHELIAL_CELL_PROLIFERATION(GO-0050680) | | 45 | 0.004478 |
| POSITIVE_REGULATION_OF_BLOOD_VESSEL_ENDOTHELIAL_CELL_MIGRATION(GO-0043536) | | 17 | 0.004567 |
| REGULATION_OF_CYSTEINE-TYPE_ENDOPEPTIDASE_ACTIVITY_INVOLVED_IN_APOPTOTIC_PROCESS(GO-0043281) | | 114 | 0.004581 |
| EYE_DEVELOPMENT(GO-0001654) | | 82 | 0.004656 |
| REGULATION_OF_LEUKOCYTE_MEDIATED_CYTOTOXICITY(GO-0001910) | | 17 | 0.004827 |
| POSITIVE_REGULATION_OF_CELL_PROLIFERATION(GO-0008284) | | 328 | 0.005006 |
| SEX_DIFFERENTIATION(GO-0007548) | | 73 | 0.00511 |
| REGULATION_OF_CHEMOKINE_PRODUCTION(GO-0032642) | | 29 | 0.005241 |
| NEGATIVE_REGULATION_OF_PROTEIN_COMPLEX_ASSEMBLY(GO-0031333) | | 23 | 0.005359 |
| POSITIVE_REGULATION_OF_T_CELL_ACTIVATION(GO-0050870) | | 120 | 0.005366 |
| REGULATION_OF_T_CELL_ACTIVATION(GO-0050863) | | 145 | 0.005376 |
| POSITIVE_REGULATION_OF_IMMUNE_RESPONSE(GO-0050778) | | 230 | 0.005521 |
| REGULATION_OF_VIRAL_GENOME_REPLICATION(GO-0045069) | | 24 | 0.005642 |
| STEROID_METABOLIC_PROCESS(GO-0008202) | | 146 | 0.005735 |
| NEURON_DIFFERENTIATION(GO-0030182) | | 422 | 0.005836 |
| LEUKOCYTE_CHEMOTAXIS(GO-0030595) | | 49 | 0.005854 |
| RESPONSE_TO_CARBOHYDRATE_STIMULUS(GO-0009743) | | 30 | 0.005948 |
| GONAD_DEVELOPMENT(GO-0008406) | | 53 | 0.005983 |
| PROTEIN_N-LINKED_GLYCOSYLATION_VIA_ASPARAGINE(GO-0018279) | | 77 | 0.006039 |
| APOPTOTIC_MITOCHONDRIAL_CHANGES(GO-0008637) | | 18 | 0.006122 |
| POSITIVE_REGULATION_OF_PROTEIN_CATABOLIC_PROCESS(GO-0045732) | | 47 | 0.006124 |
| GLYCOPROTEIN_BIOSYNTHETIC_PROCESS(GO-0009101) | | 166 | 0.006128 |
| TOLL-LIKE_RECEPTOR_4_SIGNALING_PATHWAY(GO-0034142) | | 72 | 0.006142 |
| CELL_DEATH(GO-0008219) | | 436 | 0.006278 |
| REGULATION_OF_PROTEIN_BINDING(GO-0043393) | | 52 | 0.006343 |
| REGULATION_OF_INSULIN_RECEPTOR_SIGNALING_PATHWAY(GO-0046626) | | 16 | 0.006905 |
| POSITIVE_REGULATION_OF_CYTOKINE_SECRETION(GO-0050715) | | 36 | 0.007057 |
| REPRODUCTIVE_STRUCTURE_DEVELOPMENT(GO-0048608) | | 61 | 0.007061 |
| ANTIGEN_PROCESSING_AND_PRESENTATION_OF_EXOGENOUS_PEPTIDE_ANTIGEN_VIA_MHC_CLASS_I__TAP-DEPENDENT(GO-0002479) | | 70 | 0.007085 |
| POSITIVE_REGULATION_OF_PROTEIN_KINASE_ACTIVITY(GO-0045860) | | 289 | 0.007144 |
| TRNA_AMINOACYLATION_FOR_PROTEIN_TRANSLATION(GO-0006418) | | 33 | 0.007199 |
| POSITIVE_REGULATION_OF_ANGIOGENESIS(GO-0045766) | | 53 | 0.007204 |
| FEMALE_PREGNANCY(GO-0007565) | | 42 | 0.007211 |
| REGULATION_OF_ANGIOGENESIS(GO-0045765) | | 97 | 0.007232 |
| PROTEIN_N-LINKED_GLYCOSYLATION(GO-0006487) | | 81 | 0.007255 |
| INTRACELLULAR_PROTEIN_KINASE_CASCADE(GO-0007243) | | 259 | 0.007375 |
| POSITIVE_REGULATION_OF_RECEPTOR-MEDIATED_ENDOCYTOSIS(GO-0048260) | | 21 | 0.007381 |
| VESICLE-MEDIATED_TRANSPORT(GO-0016192) | | 426 | 0.007383 |
| REGULATION_OF_CYSTEINE-TYPE_ENDOPEPTIDASE_ACTIVITY(GO-2000116) | | 118 | 0.007398 |
| DIGESTIVE_TRACT_MORPHOGENESIS(GO-0048546) | | 17 | 0.007405 |
| SIGNAL_TRANSDUCTION_BY_P53_CLASS_MEDIATOR_RESULTING_IN_INDUCTION_OF_APOPTOSIS(GO-0072332) | | 19 | 0.007421 |
| REGULATION_OF_BINDING(GO-0051098) | | 96 | 0.007449 |
| REGULATION_OF_ENDOTHELIAL_CELL_MIGRATION(GO-0010594) | | 54 | 0.007472 |
| NEGATIVE_REGULATION_OF_CANONICAL_WNT_RECEPTOR_SIGNALING_PATHWAY(GO-0090090) | | 54 | 0.007567 |
| NEGATIVE_REGULATION_OF_PROTEIN_BINDING(GO-0032091) | | 24 | 0.007619 |
| GLYCOSAMINOGLYCAN_METABOLIC_PROCESS(GO-0030203) | | 36 | 0.007633 |
| REGULATION_OF_CELLULAR_RESPONSE_TO_INSULIN_STIMULUS(GO-1900076) | | 17 | 0.007664 |
| ANTIGEN_PROCESSING_AND_PRESENTATION_OF_EXOGENOUS_PEPTIDE_ANTIGEN_VIA_MHC_CLASS_I(GO-0042590) | | 73 | 0.007776 |
| AMINE_TRANSPORT(GO-0015837) | | 87 | 0.007874 |
| REGULATION_OF_EXOCYTOSIS(GO-0017157) | | 37 | 0.007949 |
| NEGATIVE_REGULATION_OF_EPIDERMAL_GROWTH_FACTOR_RECEPTOR_SIGNALING_PATHWAY(GO-0042059) | | 35 | 0.007958 |
| PROTEIN_LIPIDATION(GO-0006497) | | 30 | 0.007987 |
| TOLL_SIGNALING_PATHWAY(GO-0008063) | | 72 | 0.007993 |
| REGULATION_OF_ACTIN_FILAMENT_POLYMERIZATION(GO-0030833) | | 26 | 0.008011 |
| CELL_PROJECTION_ORGANIZATION(GO-0030030) | | 417 | 0.008012 |
| 'DE_NOVO'_PROTEIN_FOLDING(GO-0006458) | | 33 | 0.008454 |
| SYNAPSE_ASSEMBLY(GO-0007416) | | 27 | 0.008575 |
| ANTIGEN_PROCESSING_AND_PRESENTATION(GO-0019882) | | 92 | 0.008605 |
| REGULATION_OF_DNA_DAMAGE_RESPONSE__SIGNAL_TRANSDUCTION_BY_P53_CLASS_MEDIATOR(GO-0043516) | | 20 | 0.008651 |
| NEUTROPHIL_CHEMOTAXIS(GO-0030593) | | 17 | 0.008654 |
| REGULATION_OF_BMP_SIGNALING_PATHWAY(GO-0030510) | | 32 | 0.008695 |
| ANGIOGENESIS(GO-0001525) | | 92 | 0.00873 |
| POSITIVE_REGULATION_OF_TRANSCRIPTION_FROM_RNA_POLYMERASE_II_PROMOTER(GO-0045944) | | 337 | 0.00873 |
| POSITIVE_REGULATION_OF_PEPTIDYL-SERINE_PHOSPHORYLATION(GO-0033138) | | 26 | 0.008745 |
| EYE_MORPHOGENESIS(GO-0048592) | | 44 | 0.008751 |
| PROGRAMMED_CELL_DEATH(GO-0012501) | | 415 | 0.008824 |
| POSITIVE_REGULATION_OF_KINASE_ACTIVITY(GO-0033674) | | 301 | 0.008841 |
| POSITIVE_REGULATION_OF_CELL_DEATH(GO-0010942) | | 377 | 0.009345 |
| APOPTOTIC_PROCESS(GO-0006915) | | 412 | 0.009444 |
| REGULATION_OF_PLATELET_ACTIVATION(GO-0010543) | | 16 | 0.009599 |
| NEGATIVE_REGULATION_OF_CELL_PROLIFERATION(GO-0008285) | | 301 | 0.009655 |
| REGULATION_OF_LYMPHOCYTE_MIGRATION(GO-2000401) | | 17 | 0.009667 |
| MACROMOLECULE_GLYCOSYLATION(GO-0043413) | | 147 | 0.00967 |
| TRANSFORMING_GROWTH_FACTOR_BETA_RECEPTOR_SIGNALING_PATHWAY(GO-0007179) | | 48 | 0.00983 |
| MESENCHYMAL_CELL_DIFFERENTIATION(GO-0048762) | | 46 | 0.009863 |
| POSITIVE_REGULATION_OF_MAP_KINASE_ACTIVITY(GO-0043406) | | 112 | 0.010055 |
| ACTIVATION_OF_CYSTEINE-TYPE_ENDOPEPTIDASE_ACTIVITY_INVOLVED_IN_APOPTOTIC_PROCESS(GO-0006919) | | 61 | 0.010197 |
| POSITIVE_REGULATION_OF_TRANSFERASE_ACTIVITY(GO-0051347) | | 307 | 0.010392 |
| REGULATION_OF_CELLULAR_LOCALIZATION(GO-0060341) | | 338 | 0.010675 |
| TRNA_AMINOACYLATION(GO-0043039) | | 34 | 0.010693 |
| PHOSPHOLIPID_TRANSPORT(GO-0015914) | | 15 | 0.010865 |
| LONG-CHAIN_FATTY-ACYL-COA_BIOSYNTHETIC_PROCESS(GO-0035338) | | 17 | 0.011223 |
| REGULATION_OF_T_CELL_DIFFERENTIATION(GO-0045580) | | 39 | 0.011229 |
| NEGATIVE_REGULATION_OF_PROTEIN_PHOSPHORYLATION(GO-0001933) | | 36 | 0.011566 |
| VESICLE_LOCALIZATION(GO-0051648) | | 45 | 0.011593 |
| NEGATIVE_REGULATION_OF_WNT_RECEPTOR_SIGNALING_PATHWAY(GO-0030178) | | 71 | 0.011605 |
| MYD88-INDEPENDENT_TOLL-LIKE_RECEPTOR_SIGNALING_PATHWAY(GO-0002756) | | 62 | 0.011688 |
| REGULATION_OF_PROTEIN_LOCALIZATION(GO-0032880) | | 195 | 0.011735 |
| ENDOCRINE_PANCREAS_DEVELOPMENT(GO-0031018) | | 26 | 0.011744 |
| REGULATION_OF_ACTIN_CYTOSKELETON_ORGANIZATION(GO-0032956) | | 86 | 0.011756 |
| VISUAL_PERCEPTION(GO-0007601) | | 90 | 0.011778 |
| PANCREAS_DEVELOPMENT(GO-0031016) | | 30 | 0.012594 |
| MAPK_CASCADE(GO-0000165) | | 134 | 0.012596 |
| PROTEIN_GLYCOSYLATION(GO-0006486) | | 146 | 0.012609 |
| VACUOLE_ORGANIZATION(GO-0007033) | | 28 | 0.012633 |
| GPI_ANCHOR_BIOSYNTHETIC_PROCESS(GO-0006506) | | 26 | 0.01287 |
| METANEPHROS_DEVELOPMENT(GO-0001656) | | 36 | 0.012897 |
| CELL_MATURATION(GO-0048469) | | 25 | 0.012912 |
| NEGATIVE_REGULATION_OF_PHOSPHORYLATION(GO-0042326) | | 50 | 0.012931 |
| NEGATIVE_REGULATION_OF_PROTEIN_SECRETION(GO-0050709) | | 23 | 0.012993 |
| POSITIVE_REGULATION_OF_INTERFERON-GAMMA_PRODUCTION(GO-0032729) | | 17 | 0.013068 |
| POSITIVE_REGULATION_OF_SMOOTH_MUSCLE_CELL_PROLIFERATION(GO-0048661) | | 18 | 0.013217 |
| DNA_DAMAGE_RESPONSE__SIGNAL_TRANSDUCTION_RESULTING_IN_INDUCTION_OF_APOPTOSIS(GO-0008630) | | 25 | 0.013246 |
| REGULATION_OF_CELL_GROWTH(GO-0001558) | | 129 | 0.013282 |
| POSITIVE_REGULATION_OF_ANTI-APOPTOSIS(GO-0045768) | | 19 | 0.013309 |
| G1-S_TRANSITION_CHECKPOINT(GO-0071779) | | 77 | 0.013594 |
| TOLL-LIKE_RECEPTOR_3_SIGNALING_PATHWAY(GO-0034138) | | 59 | 0.013596 |
| POSITIVE_REGULATION_OF_LIPID_CATABOLIC_PROCESS(GO-0050996) | | 16 | 0.013599 |
| OSTEOBLAST_DIFFERENTIATION(GO-0001649) | | 30 | 0.013621 |
| REGULATION_OF_RECEPTOR_ACTIVITY(GO-0010469) | | 48 | 0.013644 |
| CELLULAR_SENESCENCE(GO-0090398) | | 20 | 0.013674 |
| POSITIVE_REGULATION_OF_MAPK_CASCADE(GO-0043410) | | 101 | 0.013756 |
| REGULATION_OF_EMBRYONIC_DEVELOPMENT(GO-0045995) | | 34 | 0.013788 |
| POSITIVE_REGULATION_OF_NEUROGENESIS(GO-0050769) | | 43 | 0.013789 |
| 'DE_NOVO'_POSTTRANSLATIONAL_PROTEIN_FOLDING(GO-0051084) | | 29 | 0.013813 |
| EXOCYTOSIS(GO-0006887) | | 123 | 0.013929 |
| VESICLE_ORGANIZATION(GO-0016050) | | 41 | 0.013939 |
| POSITIVE_REGULATION_OF_PROTEOLYSIS(GO-0045862) | | 47 | 0.014148 |
| EMBRYONIC_ORGAN_DEVELOPMENT(GO-0048568) | | 73 | 0.014304 |
| SENSORY_PERCEPTION_OF_LIGHT_STIMULUS(GO-0050953) | | 90 | 0.014331 |
| REGULATION_OF_WNT_RECEPTOR_SIGNALING_PATHWAY(GO-0030111) | | 110 | 0.014444 |
| BRANCHING_INVOLVED_IN_URETERIC_BUD_MORPHOGENESIS(GO-0001658) | | 17 | 0.014445 |
| CHOLESTEROL_EFFLUX(GO-0033344) | | 20 | 0.014452 |
| FEMALE_GONAD_DEVELOPMENT(GO-0008585) | | 15 | 0.014483 |
| JAK-STAT_CASCADE(GO-0007259) | | 43 | 0.014683 |
| NEGATIVE_REGULATION_OF_CELL-SUBSTRATE_ADHESION(GO-0010812) | | 20 | 0.014716 |
| SIGNAL_TRANSDUCTION_BY_P53_CLASS_MEDIATOR(GO-0072331) | | 92 | 0.014809 |
| POSITIVE_REGULATION_OF_ORGANELLE_ORGANIZATION(GO-0010638) | | 127 | 0.014856 |
| CELLULAR_RESPONSE_TO_STARVATION(GO-0009267) | | 36 | 0.014887 |
| EPITHELIAL_CELL_DIFFERENTIATION(GO-0030855) | | 79 | 0.014971 |
| RETROGRADE_VESICLE-MEDIATED_TRANSPORT__GOLGI_TO_ER(GO-0006890) | | 18 | 0.014977 |
| NEGATIVE_REGULATION_OF_SECRETION(GO-0051048) | | 51 | 0.015229 |
| EMBRYONIC_SKELETAL_SYSTEM_DEVELOPMENT(GO-0048706) | | 26 | 0.015305 |
| AMINE_METABOLIC_PROCESS(GO-0009308) | | 330 | 0.015318 |
| REGULATION_OF_NEURON_PROJECTION_DEVELOPMENT(GO-0010975) | | 85 | 0.015521 |
| REGULATION_OF_AXONOGENESIS(GO-0050770) | | 40 | 0.015684 |
| POSITIVE_REGULATION_OF_ENDOPEPTIDASE_ACTIVITY(GO-0010950) | | 77 | 0.015713 |
| BLOOD_VESSEL_DEVELOPMENT(GO-0001568) | | 144 | 0.015717 |
| REGULATION_OF_ADAPTIVE_IMMUNE_RESPONSE(GO-0002819) | | 43 | 0.015732 |
| SERINE_FAMILY_AMINO_ACID_METABOLIC_PROCESS(GO-0009069) | | 17 | 0.015761 |
| STEROID_BIOSYNTHETIC_PROCESS(GO-0006694) | | 73 | 0.01582 |
| SENSORY_PERCEPTION(GO-0007600) | | 175 | 0.015905 |
| MITOTIC_CELL_CYCLE_G1-S_TRANSITION_CHECKPOINT(GO-0031575) | | 75 | 0.016115 |
| BLOOD_VESSEL_MORPHOGENESIS(GO-0048514) | | 125 | 0.016271 |
| CARTILAGE_DEVELOPMENT(GO-0051216) | | 39 | 0.016272 |
| GLYCOSAMINOGLYCAN_BIOSYNTHETIC_PROCESS(GO-0006024) | | 26 | 0.016315 |
| REGULATION_OF_MORPHOGENESIS_OF_A_BRANCHING_STRUCTURE(GO-0060688) | | 19 | 0.016352 |
| PROTEIN_FOLDING(GO-0006457) | | 82 | 0.016429 |
| NEGATIVE_REGULATION_OF_MAP_KINASE_ACTIVITY(GO-0043407) | | 39 | 0.016564 |
| DEFENSE_RESPONSE_TO_BACTERIUM(GO-0042742) | | 39 | 0.016592 |
| NEGATIVE_REGULATION_OF_NF-KAPPAB_TRANSCRIPTION_FACTOR_ACTIVITY(GO-0032088) | | 38 | 0.01669 |
| TRANSMEMBRANE_RECEPTOR_PROTEIN_SERINE-THREONINE_KINASE_SIGNALING_PATHWAY(GO-0007178) | | 79 | 0.016873 |
| REGULATION_OF_PROTEIN_IMPORT_INTO_NUCLEUS(GO-0042306) | | 70 | 0.016928 |
| WOUND_HEALING(GO-0042060) | | 458 | 0.016972 |
| CELLULAR_RESPONSE_TO_TUMOR_NECROSIS_FACTOR(GO-0071356) | | 38 | 0.016993 |
| ACUTE_INFLAMMATORY_RESPONSE(GO-0002526) | | 18 | 0.017686 |
| LEUKOCYTE_CELL-CELL_ADHESION(GO-0007159) | | 22 | 0.017694 |
| REGULATION_OF_BODY_FLUID_LEVELS(GO-0050878) | | 455 | 0.017726 |
| REGULATION_OF_ACTIN_FILAMENT-BASED_PROCESS(GO-0032970) | | 90 | 0.017753 |
| REGULATION_OF_TYPE_I_INTERFERON-MEDIATED_SIGNALING_PATHWAY(GO-0060338) | | 29 | 0.017834 |
| REGULATION_OF_PHAGOCYTOSIS(GO-0050764) | | 18 | 0.018049 |
| UROGENITAL_SYSTEM_DEVELOPMENT(GO-0001655) | | 96 | 0.018068 |
| REGULATION_OF_PROTEIN_COMPLEX_DISASSEMBLY(GO-0043244) | | 28 | 0.018183 |
| EPITHELIAL_TO_MESENCHYMAL_TRANSITION(GO-0001837) | | 26 | 0.018529 |
| POSITIVE_REGULATION_OF_CYSTEINE-TYPE_ENDOPEPTIDASE_ACTIVITY_INVOLVED_IN_APOPTOTIC_PROCESS(GO-0043280) | | 73 | 0.018563 |
| POSITIVE_REGULATION_OF_BINDING(GO-0051099) | | 41 | 0.018567 |
| NEGATIVE_REGULATION_OF_CYSTEINE-TYPE_ENDOPEPTIDASE_ACTIVITY_INVOLVED_IN_APOPTOTIC_PROCESS(GO-0043154) | | 39 | 0.018607 |
| POSITIVE_REGULATION_OF_FIBROBLAST_PROLIFERATION(GO-0048146) | | 23 | 0.018635 |
| REGULATION_OF_INTRACELLULAR_PROTEIN_TRANSPORT(GO-0033157) | | 83 | 0.018669 |
| REGULATION_OF_PROTEIN_COMPLEX_ASSEMBLY(GO-0043254) | | 90 | 0.018718 |
| IRON_ION_TRANSPORT(GO-0006826) | | 37 | 0.01883 |
| REGULATION_OF_FAT_CELL_DIFFERENTIATION(GO-0045598) | | 26 | 0.019008 |
| GPI_ANCHOR_METABOLIC_PROCESS(GO-0006505) | | 27 | 0.019319 |
| OSSIFICATION(GO-0001503) | | 58 | 0.019507 |
| CAMERA-TYPE_EYE_DEVELOPMENT(GO-0043010) | | 60 | 0.019511 |
| UNSATURATED_FATTY_ACID_BIOSYNTHETIC_PROCESS(GO-0006636) | | 27 | 0.019526 |
| RESPONSE_TO_STARVATION(GO-0042594) | | 38 | 0.019548 |
| REGULATION_OF_EPITHELIAL_CELL_MIGRATION(GO-0010632) | | 16 | 0.019571 |
| CENTRAL_NERVOUS_SYSTEM_DEVELOPMENT(GO-0007417) | | 206 | 0.01961 |
| CHOLESTEROL_HOMEOSTASIS(GO-0042632) | | 37 | 0.019805 |
| BLOOD_COAGULATION(GO-0007596) | | 421 | 0.019867 |
| POSITIVE_REGULATION_OF_PROTEIN_SERINE-THREONINE_KINASE_ACTIVITY(GO-0071902) | | 140 | 0.02022 |
| TRIF-DEPENDENT_TOLL-LIKE_RECEPTOR_SIGNALING_PATHWAY(GO-0035666) | | 58 | 0.020235 |
| RENAL_SYSTEM_DEVELOPMENT(GO-0072001) | | 91 | 0.020267 |
| SIGNAL_TRANSDUCTION_IN_RESPONSE_TO_DNA_DAMAGE(GO-0042770) | | 101 | 0.020413 |
| CAMERA-TYPE_EYE_MORPHOGENESIS(GO-0048593) | | 33 | 0.020423 |
| LYMPHOCYTE_MIGRATION(GO-0072676) | | 15 | 0.020425 |
| POSITIVE_REGULATION_OF_HYDROLASE_ACTIVITY(GO-0051345) | | 377 | 0.02064 |
| REGULATION_OF_G-PROTEIN_COUPLED_RECEPTOR_PROTEIN_SIGNALING_PATHWAY(GO-0008277) | | 52 | 0.020741 |
| EMBRYONIC_ORGAN_MORPHOGENESIS(GO-0048562) | | 46 | 0.020803 |
| STEROL_HOMEOSTASIS(GO-0055092) | | 37 | 0.020955 |
| POSITIVE_REGULATION_OF_CELL_GROWTH(GO-0030307) | | 47 | 0.021044 |
| C21-STEROID_HORMONE_METABOLIC_PROCESS(GO-0008207) | | 22 | 0.021544 |
| REGULATION_OF_CATENIN_IMPORT_INTO_NUCLEUS(GO-0035412) | | 15 | 0.021737 |
| NEGATIVE_REGULATION_OF_G-PROTEIN_COUPLED_RECEPTOR_PROTEIN_SIGNALING_PATHWAY(GO-0045744) | | 21 | 0.021744 |
| POSITIVE_REGULATION_OF_EPITHELIAL_TO_MESENCHYMAL_TRANSITION(GO-0010718) | | 19 | 0.021803 |
| MRNA_CAPPING(GO-0006370) | | 28 | 0.021852 |
| NEGATIVE_REGULATION_OF_MAPK_CASCADE(GO-0043409) | | 42 | 0.022115 |
| POSITIVE_REGULATION_OF_GROWTH(GO-0045927) | | 63 | 0.022181 |
| INDUCTION_OF_APOPTOSIS_BY_INTRACELLULAR_SIGNALS(GO-0008629) | | 67 | 0.022183 |
| REGULATION_OF_OXIDOREDUCTASE_ACTIVITY(GO-0051341) | | 54 | 0.022184 |
| PLATELET_ACTIVATION(GO-0030168) | | 187 | 0.022188 |
| REGULATION_OF_CANONICAL_WNT_RECEPTOR_SIGNALING_PATHWAY(GO-0060828) | | 80 | 0.0222 |
| DEVELOPMENTAL_PROCESS_INVOLVED_IN_REPRODUCTION(GO-0003006) | | 117 | 0.022205 |
| C-TERMINAL_PROTEIN_LIPIDATION(GO-0006501) | | 23 | 0.022215 |
| CELL-CELL_JUNCTION_ORGANIZATION(GO-0045216) | | 84 | 0.022232 |
| POSITIVE_REGULATION_OF_ADAPTIVE_IMMUNE_RESPONSE(GO-0002821) | | 21 | 0.022264 |
| CELL_PROLIFERATION(GO-0008283) | | 307 | 0.022325 |
| VASCULATURE_DEVELOPMENT(GO-0001944) | | 155 | 0.022368 |
| HEMOSTASIS(GO-0007599) | | 425 | 0.022432 |
| CALCIUM-MEDIATED_SIGNALING(GO-0019722) | | 30 | 0.02266 |
| OVULATION_CYCLE(GO-0042698) | | 17 | 0.023653 |
| LYSOSOME_ORGANIZATION(GO-0007040) | | 18 | 0.02366 |
| POSITIVE_REGULATION_OF_INTERLEUKIN-12_PRODUCTION(GO-0032735) | | 16 | 0.023886 |
| CELL_ADHESION(GO-0007155) | | 251 | 0.023898 |
| CARBOHYDRATE_METABOLIC_PROCESS(GO-0005975) | | 384 | 0.024059 |
| ANATOMICAL_STRUCTURE_FORMATION_INVOLVED_IN_MORPHOGENESIS(GO-0048646) | | 234 | 0.02407 |
| LEARNING(GO-0007612) | | 21 | 0.024492 |
| ER_TO_GOLGI_VESICLE-MEDIATED_TRANSPORT(GO-0006888) | | 21 | 0.024586 |
| POSITIVE_REGULATION_OF_PEPTIDASE_ACTIVITY(GO-0010952) | | 82 | 0.024728 |
| POSITIVE_REGULATION_OF_WNT_RECEPTOR_SIGNALING_PATHWAY(GO-0030177) | | 39 | 0.024729 |
| NEGATIVE_REGULATION_OF_CELLULAR_PROTEIN_METABOLIC_PROCESS(GO-0032269) | | 185 | 0.02476 |
| DENDRITE_DEVELOPMENT(GO-0016358) | | 22 | 0.024764 |
| POSITIVE_REGULATION_OF_ENDOTHELIAL_CELL_MIGRATION(GO-0010595) | | 36 | 0.0248 |
| VESICLE_TARGETING(GO-0006903) | | 27 | 0.024837 |
| PLATELET_DEGRANULATION(GO-0002576) | | 79 | 0.024844 |
| PROTEIN_TRIMERIZATION(GO-0070206) | | 17 | 0.024863 |
| POSITIVE_REGULATION_OF_PROTEASOMAL_UBIQUITIN-DEPENDENT_PROTEIN_CATABOLIC_PROCESS(GO-0032436) | | 26 | 0.024943 |
| RESPONSE_TO_RETINOIC_ACID(GO-0032526) | | 44 | 0.025057 |
| CHOLESTEROL_BIOSYNTHETIC_PROCESS(GO-0006695) | | 24 | 0.025185 |
| REGULATION_OF_LYMPHOCYTE_DIFFERENTIATION(GO-0045619) | | 44 | 0.025498 |
| REGULATION_OF_PROTEIN_TYROSINE_KINASE_ACTIVITY(GO-0061097) | | 34 | 0.025878 |
| NEGATIVE_REGULATION_OF_PROTEIN_SERINE-THREONINE_KINASE_ACTIVITY(GO-0071901) | | 56 | 0.025891 |
| AMINE_BIOSYNTHETIC_PROCESS(GO-0009309) | | 74 | 0.026101 |
| REGULATION_OF_PROTEASOMAL_PROTEIN_CATABOLIC_PROCESS(GO-0061136) | | 46 | 0.026355 |
| NEGATIVE_REGULATION_OF_CYSTEINE-TYPE_ENDOPEPTIDASE_ACTIVITY(GO-2000117) | | 41 | 0.026398 |
| POSITIVE_REGULATION_OF_CELL-CELL_ADHESION(GO-0022409) | | 17 | 0.026432 |
| PROSTAGLANDIN_METABOLIC_PROCESS(GO-0006693) | | 15 | 0.026461 |
| POLYSACCHARIDE_METABOLIC_PROCESS(GO-0005976) | | 63 | 0.026503 |
| REGULATION_OF_INTERFERON-BETA_PRODUCTION(GO-0032648) | | 19 | 0.026514 |
| ICOSANOID_BIOSYNTHETIC_PROCESS(GO-0046456) | | 24 | 0.02673 |
| DETECTION_OF_BIOTIC_STIMULUS(GO-0009595) | | 18 | 0.027231 |
| REGULATION_OF_EPIDERMAL_GROWTH_FACTOR_RECEPTOR_SIGNALING_PATHWAY(GO-0042058) | | 51 | 0.02729 |
| TRIGLYCERIDE_BIOSYNTHETIC_PROCESS(GO-0019432) | | 28 | 0.027321 |
| POSITIVE_REGULATION_OF_PROTEIN_METABOLIC_PROCESS(GO-0051247) | | 394 | 0.027359 |
| NUCLEOTIDE-BINDING_DOMAIN__LEUCINE_RICH_REPEAT_CONTAINING_RECEPTOR_SIGNALING_PATHWAY(GO-0035872) | | 39 | 0.0274 |
| BRAIN_DEVELOPMENT(GO-0007420) | | 124 | 0.027418 |
| POSITIVE_REGULATION_OF_PHOSPHORYLATION(GO-0042327) | | 175 | 0.027534 |
| PROTEOGLYCAN_METABOLIC_PROCESS(GO-0006029) | | 24 | 0.027998 |
| REGULATION_OF_OSTEOCLAST_DIFFERENTIATION(GO-0045670) | | 18 | 0.028043 |
| REGULATION_OF_NEURON_APOPTOSIS(GO-0043523) | | 55 | 0.028047 |
| NEGATIVE_REGULATION_OF_PROTEOLYSIS(GO-0045861) | | 18 | 0.02817 |
| POST-GOLGI_VESICLE-MEDIATED_TRANSPORT(GO-0006892) | | 54 | 0.028174 |
| SKELETAL_SYSTEM_MORPHOGENESIS(GO-0048705) | | 39 | 0.028194 |
| REGULATION_OF_CELL_SHAPE(GO-0008360) | | 50 | 0.02886 |
| REGULATION_OF_ERK1_AND_ERK2_CASCADE(GO-0070372) | | 76 | 0.02888 |
| GOLGI_ORGANIZATION(GO-0007030) | | 22 | 0.02894 |
| REGULATION_OF_PHOSPHATIDYLINOSITOL_3-KINASE_CASCADE(GO-0014066) | | 35 | 0.029566 |
| MYELOID_CELL_DIFFERENTIATION(GO-0030099) | | 58 | 0.029707 |
| CELL_JUNCTION_ASSEMBLY(GO-0034329) | | 110 | 0.030249 |
| REGULATION_OF_NUCLEOCYTOPLASMIC_TRANSPORT(GO-0046822) | | 83 | 0.030673 |
| BONE_MORPHOGENESIS(GO-0060349) | | 18 | 0.030827 |
| CELLULAR_HOMEOSTASIS(GO-0019725) | | 297 | 0.030963 |
| REGULATION_OF_VIRAL_TRANSCRIPTION(GO-0046782) | | 62 | 0.031008 |
| SYNAPSE_ORGANIZATION(GO-0050808) | | 40 | 0.031353 |
| KIDNEY_DEVELOPMENT(GO-0001822) | | 87 | 0.031399 |
| NUCLEAR_IMPORT(GO-0051170) | | 49 | 0.031483 |
| REGULATION_OF_FIBROBLAST_PROLIFERATION(GO-0048145) | | 37 | 0.031684 |
| ACTIN_FILAMENT_BUNDLE_ASSEMBLY(GO-0051017) | | 15 | 0.031741 |
| MYD88-DEPENDENT_TOLL-LIKE_RECEPTOR_SIGNALING_PATHWAY(GO-0002755) | | 69 | 0.032163 |
| HORMONE_BIOSYNTHETIC_PROCESS(GO-0042446) | | 27 | 0.032193 |
| NUCLEOTIDE_METABOLIC_PROCESS(GO-0009117) | | 308 | 0.032642 |
| CYTOKINE_PRODUCTION(GO-0001816) | | 34 | 0.032644 |
| CELLULAR_MEMBRANE_FUSION(GO-0006944) | | 35 | 0.032707 |
| GLIOGENESIS(GO-0042063) | | 27 | 0.032714 |
| DNA_DAMAGE_RESPONSE__SIGNAL_TRANSDUCTION_BY_P53_CLASS_MEDIATOR(GO-0030330) | | 86 | 0.032919 |
| LEARNING_OR_MEMORY(GO-0007611) | | 38 | 0.033189 |
| NEGATIVE_REGULATION_OF_BINDING(GO-0051100) | | 43 | 0.033447 |
| REGULATION_OF_ANTI-APOPTOSIS(GO-0045767) | | 25 | 0.03349 |
| TOLL-LIKE_RECEPTOR_2_SIGNALING_PATHWAY(GO-0034134) | | 65 | 0.033556 |
| NEGATIVE_REGULATION_OF_NEURON_PROJECTION_DEVELOPMENT(GO-0010977) | | 15 | 0.033858 |
| ALCOHOL_METABOLIC_PROCESS(GO-0006066) | | 243 | 0.033889 |
| REGULATION_OF_EPIDERMAL_GROWTH_FACTOR-ACTIVATED_RECEPTOR_ACTIVITY(GO-0007176) | | 18 | 0.034032 |
| AUTOPHAGY(GO-0006914) | | 23 | 0.034058 |
| REGULATION_OF_CHOLESTEROL_TRANSPORT(GO-0032374) | | 22 | 0.034101 |
| ION_HOMEOSTASIS(GO-0050801) | | 283 | 0.034694 |
| REGULATION_OF_EPIDERMIS_DEVELOPMENT(GO-0045682) | | 16 | 0.034739 |
| PHENOL-CONTAINING_COMPOUND_METABOLIC_PROCESS(GO-0018958) | | 19 | 0.034975 |
| NUCLEOBASE-CONTAINING_SMALL_MOLECULE_METABOLIC_PROCESS(GO-0055086) | | 356 | 0.034979 |
| NEGATIVE_REGULATION_OF_T_CELL_ACTIVATION(GO-0050868) | | 26 | 0.03504 |
| STEROL_BIOSYNTHETIC_PROCESS(GO-0016126) | | 25 | 0.03508 |
| REGULATION_OF_NEUROGENESIS(GO-0050767) | | 147 | 0.03513 |
| POSITIVE_REGULATION_OF_CYTOSKELETON_ORGANIZATION(GO-0051495) | | 58 | 0.035225 |
| CARBOXYLIC_ACID_TRANSPORT(GO-0046942) | | 120 | 0.035233 |
| TISSUE_DEVELOPMENT(GO-0009888) | | 387 | 0.035541 |
| KIDNEY_MORPHOGENESIS(GO-0060993) | | 18 | 0.03597 |
| POSTTRANSCRIPTIONAL_REGULATION_OF_GENE_EXPRESSION(GO-0010608) | | 178 | 0.036075 |
| REGULATION_OF_MITOCHONDRION_ORGANIZATION(GO-0010821) | | 24 | 0.036094 |
| NEGATIVE_REGULATION_OF_TRANSPORT(GO-0051051) | | 132 | 0.036595 |
| REGULATION_OF_CELLULAR_AMINO_ACID_METABOLIC_PROCESS(GO-0006521) | | 53 | 0.038192 |
| NEGATIVE_REGULATION_OF_PROTEIN_COMPLEX_DISASSEMBLY(GO-0043242) | | 18 | 0.038225 |
| MITOTIC_CELL_CYCLE_G1-S_TRANSITION_DNA_DAMAGE_CHECKPOINT(GO-0031571) | | 69 | 0.038696 |
| I-KAPPAB_KINASE-NF-KAPPAB_CASCADE(GO-0007249) | | 31 | 0.039494 |
| LONG-CHAIN_FATTY-ACYL-COA_METABOLIC_PROCESS(GO-0035336) | | 19 | 0.039504 |
| REGULATION_OF_SMOOTH_MUSCLE_CELL_PROLIFERATION(GO-0048660) | | 32 | 0.03983 |
| RESPIRATORY_SYSTEM_DEVELOPMENT(GO-0060541) | | 31 | 0.040057 |
| HUMORAL_IMMUNE_RESPONSE(GO-0006959) | | 53 | 0.040079 |
| C21-STEROID_HORMONE_BIOSYNTHETIC_PROCESS(GO-0006700) | | 16 | 0.040223 |
| TOLL-LIKE_RECEPTOR_1_SIGNALING_PATHWAY(GO-0034130) | | 65 | 0.040259 |
| NEGATIVE_REGULATION_OF_CYTOKINE_BIOSYNTHETIC_PROCESS(GO-0042036) | | 16 | 0.040472 |
| CELLULAR_AMINO_ACID_METABOLIC_PROCESS(GO-0006520) | | 239 | 0.040973 |
| CELLULAR_RESPONSE_TO_INORGANIC_SUBSTANCE(GO-0071241) | | 43 | 0.041663 |
| CELLULAR_CATION_HOMEOSTASIS(GO-0030003) | | 190 | 0.041698 |
| POSITIVE_REGULATION_OF_CELL_CYCLE(GO-0045787) | | 72 | 0.042063 |
| MALE_SEX_DIFFERENTIATION(GO-0046661) | | 41 | 0.042223 |
| RESPONSE_TO_HYPOXIA(GO-0001666) | | 76 | 0.042698 |
| EAR_MORPHOGENESIS(GO-0042471) | | 24 | 0.04275 |
| REGULATION_OF_PH(GO-0006885) | | 16 | 0.042819 |
| COGNITION(GO-0050890) | | 50 | 0.043292 |
| PHOSPHATIDYLINOSITOL-MEDIATED_SIGNALING(GO-0048015) | | 51 | 0.043376 |
| CELLULAR_ION_HOMEOSTASIS(GO-0006873) | | 265 | 0.04345 |
| NEGATIVE_REGULATION_OF_GROWTH(GO-0045926) | | 97 | 0.043794 |
| PHAGOCYTOSIS(GO-0006909) | | 32 | 0.044561 |
| CELLULAR_RESPONSE_TO_NUTRIENT(GO-0031670) | | 41 | 0.045309 |
| VITAMIN_METABOLIC_PROCESS(GO-0006766) | | 84 | 0.045388 |
| NEGATIVE_REGULATION_OF_ANGIOGENESIS(GO-0016525) | | 37 | 0.045428 |
| REGULATION_OF_RESPONSE_TO_INTERFERON-GAMMA(GO-0060330) | | 20 | 0.04602 |
| RESPONSE_TO_ABIOTIC_STIMULUS(GO-0009628) | | 239 | 0.046075 |
| ODONTOGENESIS(GO-0042476) | | 25 | 0.046322 |
| GTP_CATABOLIC_PROCESS(GO-0006184) | | 121 | 0.046331 |
| NEGATIVE_REGULATION_OF_ENDOTHELIAL_CELL_MIGRATION(GO-0010596) | | 18 | 0.046343 |
| DEVELOPMENTAL_GROWTH_INVOLVED_IN_MORPHOGENESIS(GO-0060560) | | 22 | 0.046376 |
| POSITIVE_REGULATION_OF_T_CELL_DIFFERENTIATION(GO-0045582) | | 25 | 0.046389 |
| HEMOPOIESIS(GO-0030097) | | 137 | 0.046859 |
| NEGATIVE_REGULATION_OF_I-KAPPAB_KINASE-NF-KAPPAB_CASCADE(GO-0043124) | | 15 | 0.047071 |
| NEGATIVE_REGULATION_OF_INFLAMMATORY_RESPONSE(GO-0050728) | | 16 | 0.047155 |
| POSITIVE_REGULATION_OF_TRANSLATION(GO-0045727) | | 28 | 0.047416 |
| REGULATION_OF_DEFENSE_RESPONSE_TO_VIRUS_BY_VIRUS(GO-0050690) | | 28 | 0.04762 |
| RESPONSE_TO_METAL_ION(GO-0010038) | | 86 | 0.047936 |
| POSITIVE_REGULATION_OF_CELL_DIFFERENTIATION(GO-0045597) | | 225 | 0.048051 |
| INNER_EAR_DEVELOPMENT(GO-0048839) | | 25 | 0.048358 |
| ADAPTIVE_IMMUNE_RESPONSE(GO-0002250) | | 41 | 0.048849 |
| POSITIVE_REGULATION_OF_SMALL_GTPASE_MEDIATED_SIGNAL_TRANSDUCTION(GO-0051057) | | 15 | 0.04893 |
| HETEROCYCLE_METABOLIC_PROCESS(GO-0046483) | | 434 | 0.04915 |
| PHOSPHOLIPID_BIOSYNTHETIC_PROCESS(GO-0008654) | | 67 | 0.049573 |
| REGULATION_OF_ORGAN_MORPHOGENESIS(GO-2000027) | | 46 | 0.049959 |
| CELL_CYCLE_ARREST(GO-0007050) | | 90 | 0.049987 |
