# Supplementary material for: Transcriptome-Guided Functional Analyses Reveal Novel Biological Properties and Regulatory Hierarchy of Human Embryonic Stem Cell-Derived Ventricular Cardiomyocytes Crucial for Maturation
Source: PLoS One. 2013 Oct 21;8(10):e77784. doi: 10.1371/journal.pone.0077784 (PMC3804624; doi:10.1371/journal.pone.0077784)
Supplement: Table S4 — Genes co-expressed with 17 TFs. ‘17TF-merged’ indicates the number of TFs that co-expressed with any particular gene. ‘5TF-merged’ shows the number of TFs from within the core 5 TFs (ie GATA4, HAND1, NKX2.5, PPARGC1A and TCF8, highlighted in bold) that co-expressed with any particular gene. ‘% core TF’ is the proportion of TFs (out of the total 17 TFs) that belong to the core cluster of 5 TFs. For instance, LAMA4 co-expressed with 8 TFs, ie ‘17TF-merged’ is 8. It co-expressed with all 5 of the core TFs (GATA4, HAND1, NKX2.5, PPARGC1A and TCF8) ie ‘5TF-merged’ is 5. ‘% core TF’ is 5 out of 8 ie 63%. Only genes that co-expressed with 4 or 5 of the 5 core TFs are shown. (DOCX) [file pone.0077784.s004.docx]

**Table S4 Genes co-expressed with 17 TFs.** ‘17TF-merged’ indicates the number of TFs that co-expressed with any particular gene. ‘5TF-merged’ shows the number of TFs from within the core 5 TFs (ie GATA4, HAND1, NKX2.5, PPARGC1A and TCF8, highlighted in bold) that co-expressed with any particular gene. ‘% core TF’ is the proportion of TFs (out of the total 17 TFs) that belong to the core cluster of 5 TFs. For instance, LAMA4 co-expressed with 8 TFs, ie ‘17TF-merged’ is 8. It co-expressed with all 5 of the core TFs (GATA4, HAND1, NKX2.5, PPARGC1A and TCF8) ie ‘5TF-merged’ is 5. ‘% core TF’ is 5 out of 8 ie 63%. Only genes that co-expressed with 4 or 5 of the 5 core TFs are shown.

| Symbol | **GATA4** | GATA6 | **HAND1** | HAND2 | **NKX2-5** | PPARA | **PPARGC1A** | **TCF8** | MEF2C | MESP1 | SRF | TBX20 | TBX5 | IRX4 | IRX5 | ISL1 | FOXP1 | 17TF-merged | 5TF-merged | % core TF |
| --- | --- | --- | --- | --- | --- | --- | --- | --- | --- | --- | --- | --- | --- | --- | --- | --- | --- | --- | --- | --- |
| LAMA4 | **x** | x | **x** | x | **x** |  | **x** | **x** |  |  |  |  |  | x |  |  |  | 8 | 5 | 63% |
| BCAP29 | **x** |  | **x** |  | **x** |  | **x** | **x** |  |  |  |  |  | x | x |  |  | 7 | 5 | 71% |
| CAP2 | **x** |  | **x** | x | **x** |  |  | **x** |  |  |  |  |  | x | x |  |  | 7 | 4 | 57% |
| GP1BB |  | x | **x** | x | **x** |  | **x** | **x** |  |  |  |  |  | x |  |  |  | 7 | 4 | 57% |
| MICAL2 | **x** |  | **x** |  | **x** |  | **x** | **x** |  |  |  |  |  | x | x |  |  | 7 | 5 | 71% |
| PPP1R14C | **x** |  | **x** |  | **x** |  | **x** | **x** |  |  |  |  |  | x | x |  |  | 7 | 5 | 71% |
| RAGE |  | x | **x** | x | **x** |  | **x** | **x** |  |  |  |  |  | x |  |  |  | 7 | 4 | 57% |
| RBM24 | **x** |  | **x** | x | **x** |  | **x** | **x** |  |  |  |  |  | x |  |  |  | 7 | 5 | 71% |
| ZMYND11 | **x** |  |  | x | **x** |  | **x** | **x** |  |  |  |  |  | x |  |  |  | 7 | 5 | 71% |
| ARHGAP24 | **x** |  | **x** |  | **x** |  | **x** | **x** |  |  |  |  |  |  | x |  |  | 6 | 5 | 83% |
| C12ORF23 | **x** | x | **x** | x | **x** |  |  | **x** |  |  |  |  |  |  |  |  |  | 6 | 4 | 67% |
| FHL2 | **x** |  | **x** |  | **x** |  | **x** | **x** |  |  |  |  |  |  | x |  |  | 6 | 5 | 83% |
| HIST2H2BE | **x** | x | **x** |  | **x** |  | **x** |  | x |  |  |  |  |  |  |  |  | 6 | 4 | 67% |
| KLF2 | **x** |  | **x** |  | **x** |  | **x** | **x** | x |  |  |  |  |  |  |  |  | 6 | 5 | 83% |
| MSRB3 | **x** | x | **x** | x | **x** |  |  | **x** |  |  |  |  |  |  |  |  |  | 6 | 4 | 67% |
| MYH7 | **x** |  | **x** |  | **x** |  | **x** | **x** | x |  |  |  |  |  |  |  |  | 6 | 5 | 83% |
| PJA2 | **x** |  | **x** |  | **x** |  | **x** | **x** |  |  |  |  |  |  | x |  |  | 6 | 5 | 83% |
| PKP2 | **x** |  | **x** |  | **x** |  | **x** | **x** |  |  |  |  |  |  | x |  |  | 6 | 5 | 83% |
| PPP1R3C | **x** |  | **x** |  | **x** |  | **x** | **x** |  |  |  |  |  | x |  |  |  | 6 | 5 | 83% |
| PRNP | **x** |  | **x** |  | **x** |  | **x** | **x** |  |  |  |  |  |  | x |  |  | 6 | 5 | 83% |
| RASGRP3 | **x** |  | **x** |  | **x** |  | **x** | **x** |  |  |  |  |  |  | x |  |  | 6 | 5 | 83% |
| RNF103 | **x** |  | **x** |  | **x** |  | **x** | **x** |  |  |  |  |  |  | x |  |  | 6 | 5 | 83% |
| SMPX | **x** |  | **x** |  | **x** |  | **x** | **x** | x |  |  |  |  |  |  |  |  | 6 | 5 | 83% |
| SMYD1 | **x** |  | **x** |  | **x** |  | **x** | **x** |  |  |  |  |  |  | x |  |  | 6 | 5 | 83% |
| SVIL | **x** | x | **x** |  | **x** |  | **x** | **x** |  |  |  |  |  |  |  |  |  | 6 | 5 | 83% |
| TNNT2 | **x** |  | **x** |  | **x** |  | **x** | **x** | x |  |  |  |  |  |  |  |  | 6 | 5 | 83% |
| TRIM55 | **x** |  | **x** | x | **x** |  |  | **x** |  |  |  |  |  | x |  |  |  | 6 | 4 | 67% |
| UNC45B |  |  | **x** | x | **x** |  | **x** | **x** |  |  |  |  |  | x |  |  |  | 6 | 4 | 67% |
| ADCY6 | **x** |  | **x** |  | **x** |  | **x** | **x** |  |  |  |  |  |  |  |  |  | 5 | 5 | 100% |
| ATXN1 | **x** | x | **x** |  | **x** |  | **x** |  |  |  |  |  |  |  |  |  |  | 5 | 4 | 80% |
| BIVM | **x** |  | **x** |  | **x** |  | **x** | **x** |  |  |  |  |  |  |  |  |  | 5 | 5 | 100% |
| CAMK2D | **x** | x | **x** |  | **x** |  | **x** |  |  |  |  |  |  |  |  |  |  | 5 | 4 | 80% |
| CORO6 | **x** |  | **x** |  | **x** |  | **x** | **x** |  |  |  |  |  |  |  |  |  | 5 | 5 | 100% |
| CRIP2 | **x** |  | **x** |  | **x** |  | **x** | **x** |  |  |  |  |  |  |  |  |  | 5 | 5 | 100% |
| CSRP3 | **x** |  | **x** |  | **x** |  | **x** | **x** |  |  |  |  |  |  |  |  |  | 5 | 5 | 100% |
| FAM46A | **x** |  | **x** |  | **x** |  | **x** | **x** |  |  |  |  |  |  |  |  |  | 5 | 5 | 100% |
| FLJ39502 |  |  | **x** |  | **x** |  | **x** | **x** |  |  |  |  |  |  | x |  |  | 5 | 4 | 80% |
| FLJ43374 | **x** |  | **x** |  | **x** |  | **x** |  | x |  |  |  |  |  |  |  |  | 5 | 4 | 80% |
| GHR | **x** |  | **x** |  | **x** |  | **x** | **x** |  |  |  |  |  |  |  |  |  | 5 | 5 | 100% |
| GUCY1A3 | **x** |  | **x** |  | **x** |  | **x** | **x** |  |  |  |  |  |  |  |  |  | 5 | 5 | 100% |
| HSPB2 |  |  | **x** |  | **x** |  | **x** | **x** |  |  |  |  |  | x |  |  |  | 5 | 4 | 80% |
| HSPB7 | **x** |  | **x** |  | **x** |  | **x** |  | x |  |  |  |  |  |  |  |  | 5 | 4 | 80% |
| INPP4B | **x** |  | **x** |  | **x** |  | **x** | **x** |  |  |  |  |  |  |  |  |  | 5 | 5 | 100% |
| LGALS8 | **x** |  | **x** |  | **x** |  | **x** |  | x |  |  |  |  |  |  |  |  | 5 | 4 | 80% |
| MEIS1 | **x** |  | **x** |  | **x** |  |  | **x** |  |  |  |  |  |  | x |  |  | 5 | 4 | 80% |
| MOAP1 |  |  | **x** |  | **x** |  | **x** | **x** |  |  |  |  |  | x |  |  |  | 5 | 4 | 80% |
| MXRA7 | **x** |  | **x** |  | **x** |  | **x** | **x** |  |  |  |  |  |  |  |  |  | 5 | 5 | 100% |
| MYBPC3 | **x** |  | **x** |  | **x** |  | **x** |  | x |  |  |  |  |  |  |  |  | 5 | 4 | 80% |
| MYL2 | **x** |  | **x** |  | **x** |  | **x** | **x** |  |  |  |  |  |  |  |  |  | 5 | 5 | 100% |
| MYL9 | **x** | x | **x** |  | **x** |  |  | **x** |  |  |  |  |  |  |  |  |  | 5 | 4 | 80% |
| MYOM1 | **x** |  | **x** |  | **x** |  | **x** |  | x |  |  |  |  |  |  |  |  | 5 | 4 | 80% |
| NRP1 |  |  | **x** |  | **x** |  | **x** | **x** |  |  |  |  |  |  | x |  |  | 5 | 4 | 80% |
| OXR1 |  |  | **x** |  | **x** |  | **x** | **x** |  |  |  |  |  |  | x |  |  | 5 | 4 | 80% |
| POPDC2 | **x** |  | **x** |  | **x** |  | **x** | **x** |  |  |  |  |  |  |  |  |  | 5 | 5 | 100% |
| PRKAA1 | **x** |  | **x** |  | **x** |  | **x** | **x** |  |  |  |  |  |  |  |  |  | 5 | 5 | 100% |
| PRKAG2 | **x** |  | **x** |  | **x** |  | **x** | **x** |  |  |  |  |  |  |  |  |  | 5 | 5 | 100% |
| RBM20 | **x** |  | **x** |  | **x** |  | **x** | **x** |  |  |  |  |  |  |  |  |  | 5 | 5 | 100% |
| RCOR3 | **x** |  | **x** |  | **x** |  | **x** | **x** |  |  |  |  |  |  |  |  |  | 5 | 5 | 100% |
| SCMH1 |  |  | **x** |  | **x** |  | **x** | **x** |  |  |  |  |  | x |  |  |  | 5 | 4 | 80% |
| SIPA1L2 | **x** |  | **x** |  | **x** |  | **x** | **x** |  |  |  |  |  |  |  |  |  | 5 | 5 | 100% |
| SMYD2 |  |  | **x** |  | **x** |  | **x** | **x** | x |  |  |  |  |  |  |  |  | 5 | 4 | 80% |
| SORBS2 | **x** |  | **x** |  | **x** |  | **x** | **x** |  |  |  |  |  |  |  |  |  | 5 | 5 | 100% |
| TCEA3 |  |  | **x** |  | **x** |  | **x** | **x** |  |  |  |  |  |  | x |  |  | 5 | 4 | 80% |
| TMOD1 |  |  | **x** |  | **x** |  | **x** | **x** |  |  |  |  |  |  | x |  |  | 5 | 4 | 80% |
| TNNC1 | **x** |  | **x** |  | **x** |  | **x** | **x** |  |  |  |  |  |  |  |  |  | 5 | 5 | 100% |
| ZAK |  |  | **x** |  | **x** |  | **x** | **x** |  |  |  |  |  |  | x |  |  | 5 | 4 | 80% |
| ABCB4 | **x** |  | **x** |  | **x** |  | **x** |  |  |  |  |  |  |  |  |  |  | 4 | 4 | 100% |
| APOBEC2 | **x** |  | **x** |  | **x** |  | **x** |  |  |  |  |  |  |  |  |  |  | 4 | 4 | 100% |
| C4ORF18 | **x** |  | **x** |  | **x** |  |  | **x** |  |  |  |  |  |  |  |  |  | 4 | 4 | 100% |
| COMMD3 | **x** |  | **x** |  | **x** |  |  | **x** |  |  |  |  |  |  |  |  |  | 4 | 4 | 100% |
| EFEMP2 | **x** |  | **x** |  | **x** |  |  | **x** |  |  |  |  |  |  |  |  |  | 4 | 4 | 100% |
| FHOD3 | **x** |  | **x** |  | **x** |  | **x** |  |  |  |  |  |  |  |  |  |  | 4 | 4 | 100% |
| GNS | **x** |  | **x** |  | **x** |  |  | **x** |  |  |  |  |  |  |  |  |  | 4 | 4 | 100% |
| GSTK1 | **x** |  | **x** |  | **x** |  | **x** |  |  |  |  |  |  |  |  |  |  | 4 | 4 | 100% |
| GYPC | **x** |  | **x** |  | **x** |  |  | **x** |  |  |  |  |  |  |  |  |  | 4 | 4 | 100% |
| HAK | **x** |  | **x** |  | **x** |  | **x** |  |  |  |  |  |  |  |  |  |  | 4 | 4 | 100% |
| **HAND1** | **x** |  |  |  | **x** |  | **x** | **x** |  |  |  |  |  |  |  |  |  | 4 | 4 | 100% |
| HSPB3 |  |  | **x** |  | **x** |  | **x** | **x** |  |  |  |  |  |  |  |  |  | 4 | 4 | 100% |
| IGFBP5 | **x** |  | **x** |  | **x** |  |  | **x** |  |  |  |  |  |  |  |  |  | 4 | 4 | 100% |
| INPP5A | **x** |  | **x** |  | **x** |  | **x** |  |  |  |  |  |  |  |  |  |  | 4 | 4 | 100% |
| NCAM1 | **x** |  | **x** |  | **x** |  | **x** |  |  |  |  |  |  |  |  |  |  | 4 | 4 | 100% |
| NEBL | **x** |  | **x** |  | **x** |  | **x** |  |  |  |  |  |  |  |  |  |  | 4 | 4 | 100% |
| **NKX2-5** | **x** |  | **x** |  |  |  | **x** | **x** |  |  |  |  |  |  |  |  |  | 4 | 4 | 100% |
| PAM | **x** |  | **x** |  | **x** |  | **x** |  |  |  |  |  |  |  |  |  |  | 4 | 4 | 100% |
| PLN |  |  | **x** |  | **x** |  | **x** | **x** |  |  |  |  |  |  |  |  |  | 4 | 4 | 100% |
| RRAD |  |  | **x** |  | **x** |  | **x** | **x** |  |  |  |  |  |  |  |  |  | 4 | 4 | 100% |
| SMARCD3 | **x** |  | **x** |  | **x** |  | **x** |  |  |  |  |  |  |  |  |  |  | 4 | 4 | 100% |
| TPM1 | **x** |  | **x** |  | **x** |  | **x** |  |  |  |  |  |  |  |  |  |  | 4 | 4 | 100% |
