# Supplementary figures and images for: Transcriptome-Guided Functional Analyses Reveal Novel Biological Properties and Regulatory Hierarchy of Human Embryonic Stem Cell-Derived Ventricular Cardiomyocytes Crucial for Maturation
Source: PLoS One. 2013 Oct 21;8(10):e77784. doi: 10.1371/journal.pone.0077784 (PMC3804624; doi:10.1371/journal.pone.0077784)

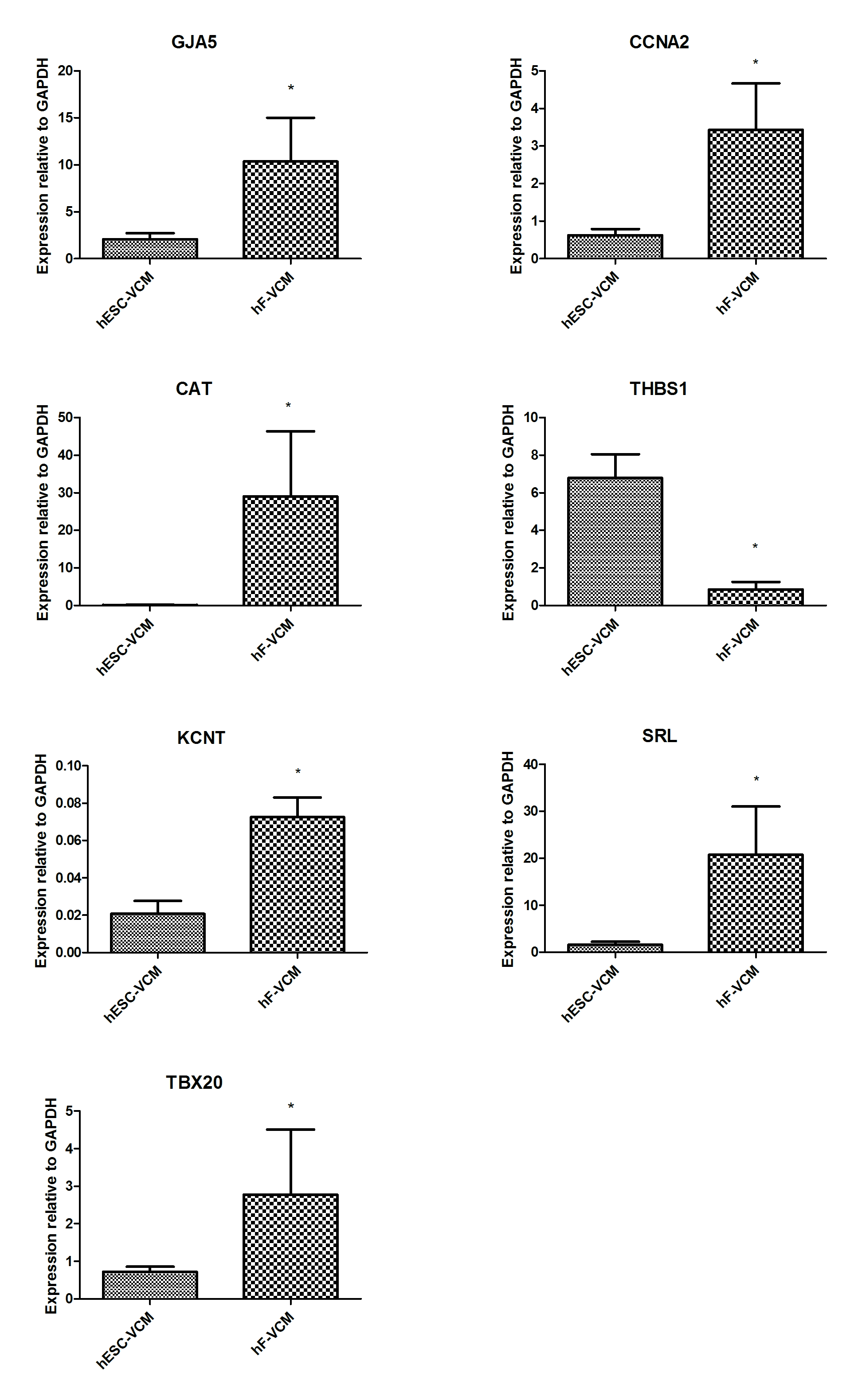

Supplement: Figure S1 — QPCR analysis of selected genes in hESC-VCMs and hF-VCMs. Expression was normalized to GAPDH. * p<0.05. (TIF) [file pone.0077784.s006.tif]
