# Supplementary material for: Transcriptome-Guided Functional Analyses Reveal Novel Biological Properties and Regulatory Hierarchy of Human Embryonic Stem Cell-Derived Ventricular Cardiomyocytes Crucial for Maturation
Source: PLoS One. 2013 Oct 21;8(10):e77784. doi: 10.1371/journal.pone.0077784 (PMC3804624; doi:10.1371/journal.pone.0077784)
Supplement: Methods S1 — Supporting methods. (DOCX) [file pone.0077784.s007.docx]

**Supplemental information**

**Methods**

**Culture of undifferentiated hESCs**

Undifferentiated HES2 (NIH code ES02) obtained from ESI International (Singapore), were maintained at 37°C and 5% CO_2_ on irradiated mouse embryonic fibroblasts in hESC media consisting of DMEM/F12 (50:50; Mediatech, Herndon, VA) supplemented with 20% knock-out serum replacement, 100 µM nonessential amino acids, 2 mM glutamine, 50 U/ml penicillin, 50 µg/ml streptomycin (Invitrogen, Grand Island, NY), 100µM β-mercaptoethanol (Sigma, St Louis, MO), and 20 ng/mL βFGF (R&D Systems, Minneapolis, MN)

**Isolation of hESC-VCMs and lentivirus (LV)-mediated gene transfer**

We differentiated hESCs into CMs using established protocols through application of growth factors at specific stages of differentiation [[1](#_ENREF_1)]. The following cytokines were used: days 0–1, BMP4 (0.5 ng/ml); days 1–4, BMP4 (10 ng/ml), βFGF (5 ng/ml) and activin A (3 ng/ml); days 4–8, DKK1 (150 ng/ml) and VEGF (10 ng/ml); after day 8, VEGF (10 ng/ml), DKK1 (150 ng/ml) and βFGF (5 ng/ml). Cultures were maintained in a 5% CO_2_/5% O_2_/90% N_2_ environment for the first 10–12 days and were then transferred into a 5% CO_2_/air environment. After approximately 12-14 days, cardiac derivatives made up ~50% of the cell population.

For profiling experiments, hESC-**V**CMs were purified using the LV-MLC2v-mCherry reporter system to avoid ambiguities due to the presence of contaminating non-ventricular CMs and non-cardiac cells. We transduced 20-30 day old hESC-derived cells with recombinant LV-MLC2v-mCherry particles at a titer equal to or greater than 10^6^ and an MOI of 3. Fluorescing mCherry positive cells became visible 72 hours post-transduction and were sorted away from the remaining cell types using flow-activated cell sorting (BD FACSAriaTM II). The efficacy of using the MLC2v promoter to drive the expression of a reporter protein for identifying **V**CMs has been described previously [[2](#_ENREF_2)].

**Isolation of hF- and A-VCMs**

Human fetal (hF) and adult (hA) left **V**CMs were isolated and experimented according to protocols approved by the UC Davis IUPAC and IRB (Protocol #200614787-1 and # 200614594-1). All fetal hearts (18-20 weeks) and adult (53-70 years) were digested using the Langendorff system with a recirculatory system that circulated the 37°C collagenase solution until cells started to dissociate into the enzyme solution. The fetal hearts typically took about 30min and the adult hearts took over 3 hours to digest. The collagenase solution had 200U/ml collagenase II (Worthington Biochemical Corp), 4mg protease (Sigma) with 1% BSA. After enzyme treatment, the hearts were chopped manually to release the cells into high K+ solution. The fetal cells were plated for 1 hour in M199 with 5mM carnitine, 5mM creatine, 5mM taurine, 10% FBS and 1% pen/strep to remove fibroblasts, then the medium was collected to retrieve the CMs still in suspension. The adult cells were not plated but allowed to settle by gravity for 15 min. The denser cardiomyocytes at the bottom of the conical tubes were collected.

**Data analysis**

Our data set consisted of 48804 probes × 10 samples, including 2 hESCs, 2 hESC-**V**CMs, 3 hF-**V**CMs, and 3 hA-**V**CMs. Expression was normalized using background subtraction and cubic spline within the BeadStudio software package (Illumina) [[3](#_ENREF_3)].

For hierarchical clustering, we used average linkage based on Euclidean distance. PCA was performed using the Matlab function princomp.

Database version: For the Gene Ontology (GO) data, we used version 1.1.2681 of the file gene ontology.1 0.obo (Time stamp: 06:03:2012 19:30, downloaded from the GO official website at http://www.geneontology.org/ontology/obo format 1 0/gene ontology.1 0.obo). For the Homo Sapiens annotation file, we used version 1.225 of the file gene association.goa human (Time stamp: 06:03:2012, downloaded from the Gene Ontology Annotation (UniProt-GOA) Database at ftp://ftp.ebi.ac.uk/pub/databases/GO/goa/HUMAN/gene association.goa human.gz). To use up-to-date gene ontology and annotation data, we constructed our Homo Sapiens Biological Process (HSBP) gene sets using a similar method as is adopted for the GSEA official MsigDB C5 gene sets (see http://www.broadinstitute.org/gsea/msigdb/collection details.jsp#C5). Specifically, only entries associated with the following evidence codes were included: IDA, IPI, IMP, IGI, IEP, ISS, and TAS. We removed gene sets with more than 500 genes or fewer than 15, to exclude very broad categories or very narrow ones, as suggested by the GSEA user guide [[4](#_ENREF_4)].

For GSEA, we used the HSBP gene set database. After normalization, about 1/4 of the expression values are negative. Specifically, the expression values of 20877 genes are all positive and 871 genes are all negative. The minimum value is -45.4700, the minimum positive value is 1.6673e-004, the maximum positive value is 1.0013e+005, and the mean positive value is 584.1625. As suggested by Dunning et al [[5](#_ENREF_5)], a small offset, which equals the sum of the absolute minimum value and the minimum positive value (i.e., 45.4700 + 1.6673e-004), was added to the data to ensure positivity. The dataset was collapsed to unique genes using the averaged values of multiple probes for the same gene. Log2 transformation was then applied. We adopted most of the default settings of GSEA. Specifically, the number of permutation is set to 1000, max size of gene sets: 500 and min size of gene sets: 15. Some modifications were made due to the small sample size of the dataset. Specifically, we used gene set permutation (rather than the default phenotype permutation and the Diff of Classes metric (rather than the default Signal2Noise metric) for ranking genes. Due to these settings, we used a more stringent FDR cutoff 5% (rather than the default threshold 25%) to identify significant gene sets, as suggested by the GSEA user manual[[4](#_ENREF_4)].

**Quantitative Real-time PCR (qRT-PCR)**

CDNA was prepared using the QuantiTect Reverse Transcription Kit (Qiagen). QRT- PCR was carried out using the Power SYBR® Green PCR Master Mix (Applied Biosystems) and gene expressions were quantified using StepOnePlusTM Real-Time PCR system (Applied Biosystems). Primer sequences are available upon request. Gene expression was normalized to GAPDH. Results are presented as mean+/-SEM. A Student’s t-test was employed to determine statistical significance. P values less than 0.05 were considered as statistically significant.

**References**

1. Yang L, MH Soonpaa, ED Adler, TK Roepke, SJ Kattman, M Kennedy, E Henckaerts, K Bonham, GW Abbott, RM Linden, LJ Field and GM Keller. (2008). Human cardiovascular progenitor cells develop from a KDR+ embryonic-stem-cell-derived population. Nature 453:524-8.

2. Fu JD, SN Rushing, DK Lieu, CW Chan, CW Kong, L Geng, KD Wilson, N Chiamvimonvat, KR Boheler, JC Wu, G Keller, RJ Hajjar and RA Li. (2011). Distinct roles of microRNA-1 and -499 in ventricular specification and functional maturation of human embryonic stem cell-derived cardiomyocytes. PLoS One 6:e27417.

3. Consortium M and L Shi and LH Reid and WD Jones and R Shippy and JA Warrington and SC Baker and PJ Collins and F de Longueville and ES Kawasaki and KY Lee and Y Luo and YA Sun and JC Willey and RA Setterquist and GM Fischer and W Tong and YP Dragan and DJ Dix and FW Frueh and FM Goodsaid and D Herman and RV Jensen and CD Johnson and EK Lobenhofer and RK Puri and U Schrf and J Thierry-Mieg and C Wang and M Wilson and PK Wolber and L Zhang and S Amur and W Bao and CC Barbacioru and AB Lucas and V Bertholet and C Boysen and B Bromley and D Brown and A Brunner and R Canales and XM Cao and TA Cebula and JJ Chen and J Cheng and TM Chu and E Chudin and J Corson and JC Corton and LJ Croner and C Davies and TS Davison and G Delenstarr and X Deng and D Dorris and AC Eklund and XH Fan and H Fang and S Fulmer-Smentek and JC Fuscoe and K Gallagher and W Ge and L Guo and X Guo and J Hager and PK Haje and J Han and T Han and HC Harbottle and SC Harris and E Hatchwell and CA Hauser and S Hester and H Hong and P Hurban and SA Jackson and H Ji and CR Knight and WP Kuo and JE LeClerc and S Levy and QZ Li and C Liu and Y Liu and MJ Lombardi and Y Ma and SR Magnuson and B Maqsodi and T McDaniel and N Mei and O Myklebost and B Ning and N Novoradovskaya and MS Orr and TW Osborn and A Papallo and TA Patterson and RG Perkins and EH Peters and R Peterson and KL Philips and PS Pine and L Pusztai and F Qian and H Ren and M Rosen and BA Rosenzweig and RR Samaha and M Schena and GP Schroth and S Shchegrova and DD Smith and F Staedtler and Z Su and H Sun and Z Szallasi and Z Tezak and D Thierry-Mieg and KL Thompson and I Tikhonova and Y Turpaz and B Vallanat and C Van and SJ Walker and SJ Wang and Y Wang and R Wolfinger and A Wong and J Wu and C Xiao and Q Xie and J Xu and W Yang and L Zhang and S Zhong and Y Zong and W Slikker, Jr. (2006). The MicroArray Quality Control (MAQC) project shows inter- and intraplatform reproducibility of gene expression measurements. Nat Biotechnol 24:1151-61.

4. Kuehn H. (2007). GSEA user guide.

5. Dunning MJ, NL Barbosa-Morais, AG Lynch, S Tavare and ME Ritchie. (2008). Statistical issues in the analysis of Illumina data. BMC Bioinformatics 9:85.
